# Fibroblast membrane-camouflaged nanoparticles for inflammation treatment in the early stage

**DOI:** 10.1038/s41368-021-00144-2

**Published:** 2021-11-16

**Authors:** Lizhong Sun, Libang He, Wei Wu, Li Luo, Mingyue Han, Yifang Liu, Shijie Shi, Kaijing Zhong, Jiaojiao Yang, Jiyao Li

**Affiliations:** 1 State Key Laboratory of Oral Diseases & National Clinical Research Center for Oral Diseases & Department of Cariology and Endodontics, West China Hospital of Stomatology, Sichuan University, Chengdu, China; 2 Key Laboratory for Biorheological Science and Technology of Ministry of Education, State and Local Joint Engineering Laboratory for Vascular Implants, Bioengineering College of Chongqing University, Chongqing, China

**Keywords:** Bacterial infection, Pulpitis

## Abstract

Unrestrained inflammation is harmful to tissue repair and regeneration. Immune cell membrane-camouflaged nanoparticles have been proven to show promise as inflammation targets and multitargeted inflammation controls in the treatment of severe inflammation. Prevention and early intervention of inflammation can reduce the risk of irreversible tissue damage and loss of function, but no cell membrane-camouflaged nanotechnology has been reported to achieve stage-specific treatment in these conditions. In this study, we investigated the prophylactic and therapeutic efficacy of fibroblast membrane-camouflaged nanoparticles for topical treatment of early inflammation (early pulpitis as the model) with the help of in-depth bioinformatics and molecular biology investigations in vitro and in vivo. Nanoparticles have been proven to act as sentinels to detect and competitively neutralize invasive *Escherichia coli* lipopolysaccharide (*E. coli* LPS) with resident fibroblasts to effectively inhibit the activation of intricate signaling pathways. Moreover, nanoparticles can alleviate the secretion of multiple inflammatory cytokines to achieve multitargeted anti-inflammatory effects, attenuating inflammatory conditions in the early stage. Our work verified the feasibility of fibroblast membrane-camouflaged nanoparticles for inflammation treatment in the early stage, which widens the potential cell types for inflammation regulation.

## Introduction

Inflammation plays a central role in the innate immunity, and it comprises a wide variety of pathological processes that are usually in response to infection. When infection occurs, host cells in the innate immune system sense the damaging insult from invading microorganisms.^1^ Then, the interaction between pathogenic microorganisms and host cells mediates inflammatory progression from the early stage to the advanced stage.^2,3^ In the early stage of inflammation, some fibroblasts always participate in the activation of the inflammatory process. For instance, pulp fibroblasts (dental pulp cells, DPCs) and intestinal fibroblasts are involved in the initiation of pulpitis and local gut inflammation, respectively.^4,5^ These tissue-resident fibroblasts can first detect the damaging insult, followed by alarmed circulating immune cells migrating to the inflamed tissues.^6^ With the advancement of inflammation, immune cells overwhelm nonimmune cells to dominate in inflammatory sites. These infiltrated nonimmune cells and immune cells are able to fulfill dedicated homeostatic functions, such as surveillance and clearance of invading pathogens, by producing a plethora of inflammatory cytokines.^7^ However, localized overproduction of inflammatory cytokines leads to uncontrolled inflammation and progressive tissue damage combined with loss of function.^8,9^

The existing anti-inflammatory therapies have been proven to be moderately effective.^10^ However, their single-target inhibition may not halt the progression of the complex inflammation process efficiently.^11,12^ Recently, cell membrane-camouflaged nanoparticles have emerged as a new promising therapeutic tool to attenuate inflammatory diseases.^12–14^ Comprising a natural host cell membrane shell and nanoparticle cores, the core-shell structure retains intact antigenic exteriors and associated biological properties inherited from source cell membranes.^15–18^ These nanoparticles can attenuate inflammation by neutralizing microorganisms, virulence, or inflammatory cytokines in a multitargeted manner.^19–21^ Among biomimetic nanoplatforms, immune cell membrane-camouflaged nanoparticles have great multitargeted therapeutic efficacy by preserving a complex of membrane receptors.^15,22,23^ For example, certain inflammatory cytokine receptor-localized macrophage membranes and neutrophil membrane-camouflaged nanoparticles have been used for neutralizing broad-spectrum inflammatory cytokines in sepsis treatment and synovial inflammation management, respectively.^19,24^ These immune cell membrane-based therapies effectively block the inflammatory response due to their counterpart-sourced immune cells playing a dominant role in the advanced stage of inflammation.^6^ In addition, it is worth noting that early intervention will help to slow the process of inflammation, strongly reducing the incidence of irreversible tissue damage and loss of function.^25^ Therefore, it is necessary to develop novel anti-inflammatory approaches to suppress inflammation in the early stage.

The recognized importance of resident fibroblasts in the early stage of inflammation may lead to some therapeutic potential of fibroblast membrane-camouflaged nanoparticles in inflammation treatment.^26,27^ In our study, an early pulpitis model was used, with a particular focus on the therapeutic role of fibroblast membrane-camouflaged nanoparticles in bacterial virulence-mediated inflammation. In early pulpitis, when bacteria progress through the enamel and dentinal tubules and reach the pulp, DPCs can act as front-line troopers to detect bacterial lipopolysaccharide (LPS) via Toll-like receptor 4 (TLR4).^28,29^ Bacterial ligands bind to DPCs and activate intricate intracellular signaling pathways, which then produce a plethora of inflammatory cytokines and amplify the inflammatory response.^10,29^

**Fig. 1:**
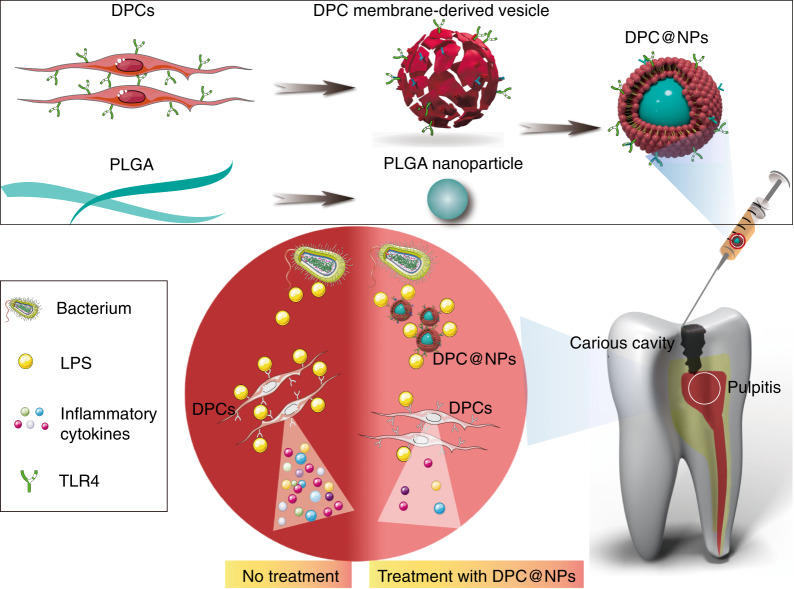
Schematic of DPC@NP fabrication and its application in the treatment of early pulpitis. DPC@NPs were fabricated by coating isolated dental pulp cell membrane-derived vesicles onto prepared PLGA nanoparticles. DPC@NPs were administered into the pulpitis tissue via injection. DPC@NPs alleviated the expression of a plethora of inflammatory cytokines to attenuate LPS-induced early pulpitis

Here, DPCs were engineered to display high expression of TLR4 antigens under an LPS stimulatory context, and the membrane of which was fused onto poly(lactic-coglycolic acid) (PLGA) nanoparticles, which can support and prevent the cell membrane from fusing and collapsing.^22,27^ Then, the fabricated novel fibroblast membrane-camouflaged nanoparticles (DPC@NPs) were investigated as a broad-spectrum anti-inflammatory agent for early inflammation regulation using high-throughput sequencing techniques, bioinformatics analysis techniques, and qualitative and quantitative analyses at the transcription and translation levels in vitro and in vivo. Binding between DPC@NPs and *E. coli* LPS led to inhibition of the expression and secretion of multiple cytokines by inactivation of some inflammation-related intracellular signaling pathways (Fig. 1). As a result, DPC@NPs were demonstrated to act as sentinels to detect the invasion of virulence factors and compete with DPCs to effectively inhibit the inflammatory process in the early stage. Overall, fibroblasts have the potential to be an ideal source for cell membrane-based nanotherapeutics in inflammation treatment, especially in early inflammation.

## Results

### Preparation and characterization of DPC@NPs

**Fig. 2:**
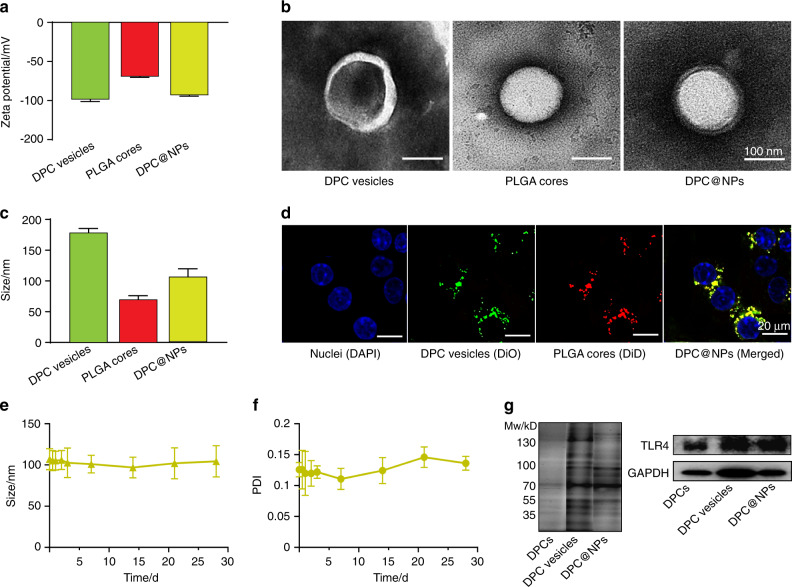
Formulation and characterization of DPC@NPs. **a** Zeta potential before and after coating of the DPC membrane onto PLGA nanoparticles. **b** Representative TEM images of DPC vesicles, PLGA cores, and DPC@NPs. **c** Diameter before and after coating of the DPC membrane onto PLGA nanoparticles. **d** CLSM images of nuclei of RAW 264.7, DiO-labeled DPC vesicles (green), DiD-labeled PLGA (red), and DPC@NPs (yellow) after internalization by RAW 264.7. **e** Size and **f** PDI stability of DPC@NPs in 1 × PBS over 28 d. **g** SDS–PAGE analysis of total proteins on the membrane of DPCs, vesicles of DPCs, and DPC@NPs (left panel) and WB analysis of the TLR4 receptor (right panel). Mw, molecular weight

The quantitative reverse transcription polymerase chain reaction (qRT–PCR) results showed that the highest messenger RNA (mRNA) level of *TLR4* in DPCs (Fig. S1) was stimulated by *E. coli* LPS at 10 μg·mL^−1^ for 6 h (Fig. S2a), resulting in the most effective activation of DPCs. This finding was further supported by flow cytometry at the protein level (Fig. S2b). Flow cytometry of TLR4-positive (+) fibroblasts was enhanced from 87.2 to 97.7% after stimulation with *E. coli* LPS at 10 μg·mL^−1^ for 6 h. The membrane from these engineered DPCs was successfully extracted, as demonstrated by staining with the lipophilic fluorescent membrane probe 3,3’-dioctadecyloxacarbocyanine perchlorate (DiO) (Fig. S2). The DPC membrane was then extruded to DPC membrane-derived vesicles. To harvest DPC@NPs, DPC membrane-derived vesicles were fused onto the surface of PLGA cores. After membrane coating, the zeta potential of DPC@NPs (−93.25 mV) was comparable with the value of pure DPC membrane-derived vesicles (−98.75 mV) but was more negative than that of unmodified PLGA cores (−69.45 mV) (Fig. 2a). The average diameter of DPC@NPs measured from DLS was approximately 107.1 ± 12.73 nm, which was ~20 nm more than that of uncoated PLGA cores (Fig. 2c and Fig. S4). The Tyndall effect of the solution containing DPC@NPs (Fig. S5) suggested the colloidal property of the biomimetic nanoparticles. These data indicated the successful fabrication of DPC@NPs. The DPC@NPs and each component were further imaged using transmission electron microscopy (TEM) after staining with phosphotungstic acid. The imaging showed a spherical morphology of pure membrane vesicles and polymeric cores as well as a characteristic “core-shell” structure after the membrane coating, which confirmed successful nanoformulation (Fig. 2b). In addition, the DPC@NPs were dual-fluorophore-labeled and internalized by a murine macrophage cell line (RAW 264.7). The resulting fluorescent images exhibited a high degree of overlap of DiO signals (green) and 1,1′-dioctadecyl-3,3,3′,3′-tetramethylindodicarbocyanine perchlorate (DiD) signals (red), which corresponded to the DPC membrane shell and PLGA core, respectively (Fig. 2d and Figs. S6–7). Furthermore, we monitored the long-term stability of DPC@NPs over time. Their size (Fig. 2e and Fig. S8) and polymer dispersity index (PDI) (Fig. 2f) remained stable for 28 days at room temperature. Thereafter, sodium dodecyl sulfate polyacrylamide gel electrophoresis (SDS–PAGE) and western blotting (WB) analysis were used to determine the presence of protein profiles and specific TLR4 after the preparation of DPC@NPs. These results clearly showed that TLR4 inherited from DPCs was retained on the vesicles of *E. coli* LPS-stimulated DPCs and DPC@NPs (Fig. 2g).

### The binding between DPC@NPs and LPS

**Fig. 3:**
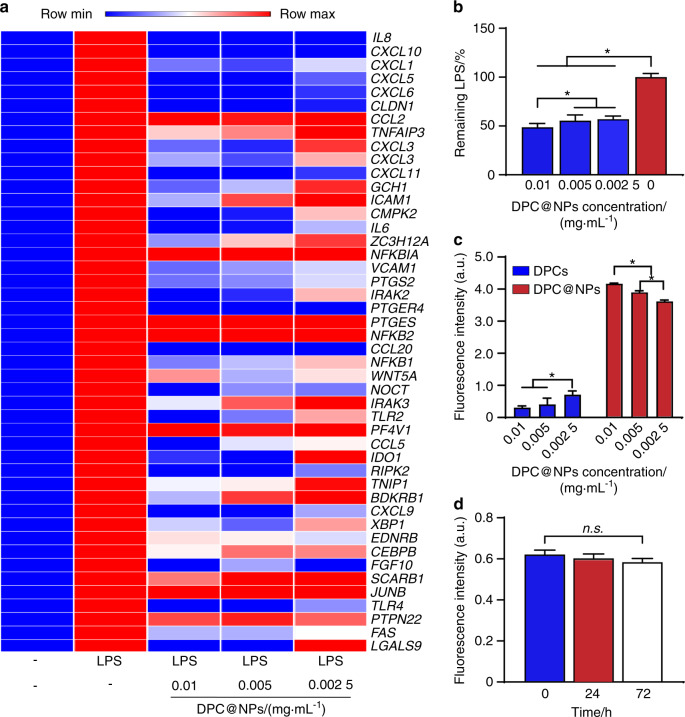
The binding of *E*. *coli* LPS to DPC@NPs. **a** Heatmap of differentially expressed genes in response to lipopolysaccharide among the control group, LPS, LPS + 0.01 mg·mL^−1^ DPC@NPs, LPS + 0.005 mg·mL^−1^ DPC@NPs, and LPS + 0.002 5 mg·mL^−1^ DPC@NPs. **b** Quantification of the *E. coli* LPS (10 μg·mL^−1^) removal with varying amounts of DPC@NPs (0.01, 0.005, and 0.002 5 mg·mL^−1^). *E. coli* LPS alone was included as a control group. **c** Competitive binding with *E. coli* LPS between various concentrations of DPC@NPs and DPCs. **d** Short-term stability of 0.01 mg·mL^−1^ DPC@NPs to neutralize *E. coli* LPS for 72 h. Statistical analyses were performed by one-way ANOVA (*P < 0.05; n.s. represents no significance). Data presented as the mean ± s.d. (*n* = 3)

Subsequently, we evaluated the functional neutralization of LPS by DPC@NPs. A Gene Ontology (GO) analysis was conducted to reveal the effect of various concentrations of DPC@NPs (0.01, 0.005, and 0.002 5 mg·mL^−1^) on the changes in the *E. coli* LPS-induced signaling pathway enrichment in the DPCs. The results showed that response to the LPS signaling pathway was the most enriched biological process (BP) term when the DPCs were stimulated with LPS alone (Fig. S9a). With the addition of various concentrations of DPC@NPs (0.01, 0.005, and 0.002 5 mg·mL^−1^), the enriched BP terms changed significantly, and the term response to the LPS signaling pathway ranked 4th (Fig. S9b), 2nd (Fig. S9c), and after the 10th (Fig. S9d) among all BP terms, respectively. In the response to the LPS signaling pathway, there were several crucial LPS-regulated genes, such as *CXCL8, CXCL10, CXCL1*, and *TLR4*. These genes were significantly activated in DPCs stimulated with *E. coli* LPS. However, with the addition of various concentrations of DPC@NPs, these genes were significantly downregulated in the DPCs (Fig. 3a).

To further reveal how DPC@NPs play an inhibitory role on *E. coli* LPS, the removal capacity of DPC@NPs was quantified.^24^ Compared with the control group containing *E. coli* LPS alone, the remaining *E. coli* LPS in the supernatant decreased when it was incubated with various concentrations of DPC@NPs (Fig. 3b). When the concentration of DPC@NPs was increased to 0.01 mg·mL^−1^, the maximal removal capacity of *E. coli* LPS was achieved. In addition, a competitive binding study showed that with an increase in the concentration of DPC@NPs, the fluorescence intensity of the supernatant, which represented fluorescein isothiocyanate (FITC)-*E. coli* LPS binding with DPC@NPs, trended upward (Fig. 3c**)**. In contrast, the fluorescence intensity of FITC-*E. coli* LPS combined with DPCs at the bottom of the six-well plate trended downward with increasing DPC@NP concentration.

In addition to functional neutralization, the stability of DPC@NPs in absorbing LPS is another key factor that should be considered. After storage of DPC@NPs for 0, 24, and 72 h, they were incubated with FITC-LPS. We collected the supernatant after centrifugation and quantified its fluorescence intensity. The fluorescence intensity in the supernatant remained stable (Fig. 3d), indicating the short-term stability of DPC@NPs to sequester *E. coli* LPS.

### Anti-inflammatory cytokine activity of DPC@NPs in vitro

**Fig. 4:**
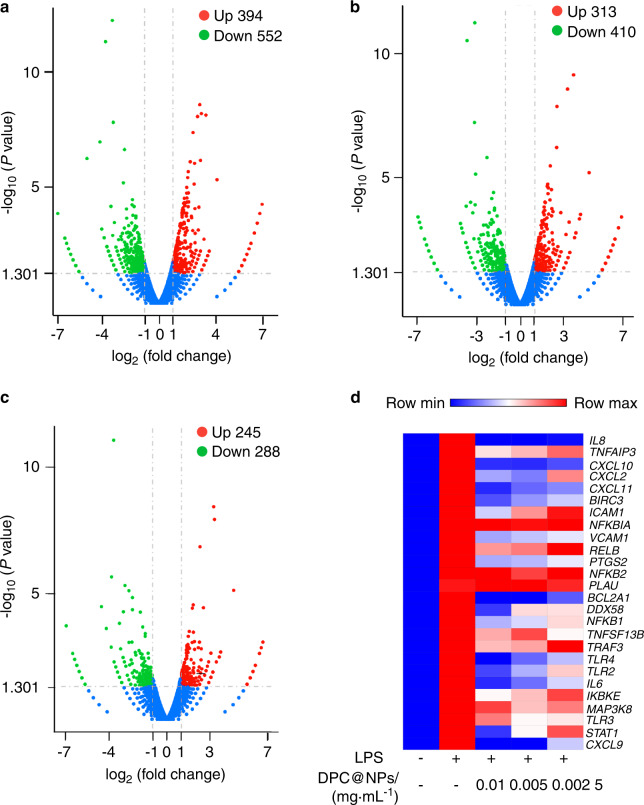
Altered expression of genes after DPC@NP treatment. Volcano map of differentially expressed genes between **a** LPS and LPS + 0.01 mg·mL^−1^ DPC@NPs, **b** LPS and LPS + 0.005 mg·mL^−1^ DPC@NPs, and **c** LPS and LPS + 0.002 5 mg·mL^−1^ DPC@NPs. **d** Heatmap of differentially expressed genes related to inflammation among the control group, LPS, LPS + 0.01 mg·mL^−1^ DPC@NPs, LPS + 0.005 mg·mL^−1^ DPC@NPs, and LPS + 0.002 5 mg·mL^−1^ DPC@NPs

To explore the broad-spectrum anti-inflammatory properties of DPC@NPs, RNA-seq was used for the total gene expression analysis. First, the total altered expression genes in each group were displayed by the volcano plot and the heatmap. In comparison with the LPS group, 394 genes were downregulated in which the DPCs were incubated with LPS + 0.01 mg·mL^−1^ DPC@NPs (Fig. 4a). When the concentrations of DPC@NPs were adjusted to 0.005 and 0.002 5 mg·mL^−1^, 313 (Fig. 4b) and 245 (Fig. 4c) downregulated genes were detected. A heatmap confirmed the alterations in gene expression among LPS, LPS + 0.01 mg·mL^−1^ DPC@NPs, LPS + 0.005 mg·mL^−1^ DPC@NPs, and LPS + 0.002 5 mg·mL^−1^ DPC@NPs (Fig. S10). Then, we further studied the effect of DPC@NPs on the expression of genes related to inflammation. The red stripe in the LPS group represents the highly expressed genes. However, blue stripes representing low expression genes were seen in the LPS + DPC@NP groups (Fig. 4d). These results signified that great genetic alterations occured under DPC@NP exposure.

**Fig. 5:**
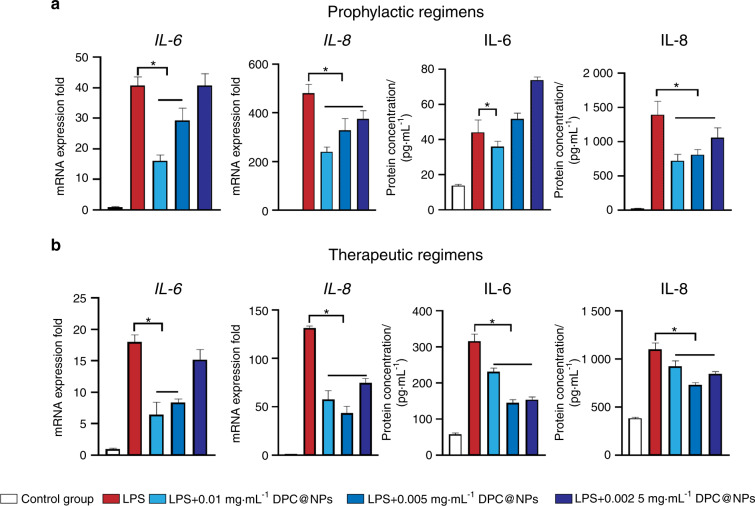
mRNA expression of *IL-6* and *IL-8* and protein secretion of IL-6 and IL-8 after DPC@NP treatment. **a** Fold change in the mRNA expression of *IL-6* and *IL-8* and protein concentrations of IL-6 and IL-8 at 24 h in the prophylactic regimens. **b** Fold change in the mRNA expression of *IL-6* and *IL-8* and protein concentration of IL-6 and IL-8 at 24 h in the therapeutic regimens. Statistical analyses were performed by one-way ANOVA (*P < 0.05). Data presented as the mean ± s.d. (*n* = 3)

Thereafter, the efficacies of DPC@NPs against the production of *interleukin-6* (*IL-6*) and *interleukin-8* (*IL-8*) were evaluated, in which DPC@NPs and *E. coli* LPS were added to the culture medium containing DPCs simultaneously as prophylactic regimens (Fig. 5a) and DPC@NPs were added after the DPCs were stimulated by *E. coli* LPS for 1 h as therapeutic regimens (Fig. 5b).^30^ The in vivo experiment showed that pulpitis was at an early stage within 24 h after LPS stimulation. Therefore, the treated DPCs were correspondingly collected at 24 h in vitro to analyze the gene expression levels of inflammatory cytokines among different groups. Compared with the control group, both *IL-6* and *IL-8* were elevated in the *E. coli* LPS-treated DPCs. This finding was in line with those of previous studies in which *E. coli* LPS was positively linked with the expression of cytokines.^10^ However, the presence of DPC@NPs allowed for lower mRNA expression of *IL-6* and *IL-8* both in the prophylactic and therapeutic regimens at 24 h. A similar inhibitory effect of DPC@NPs was also demonstrated at the protein level. Compared with *E. coli* LPS-stimulated DPCs alone, the addition of DPC@NPs greatly reduced the secretion of IL-6 and IL-8 into the supernatant from DPCs. As shown in Fig. 5, 0.01 mg·mL^−1^ DPC@NPs can always play an inhibitory role on LPS at gene and protein levels in both prophylactic and therapeutic regimens. This may be attributed to the maximum adsorption capacity of DPC@NPs at 0.01 mg·mL^−1^ for *E. coli* LPS.^24,30^

### Anti-inflammatory cytokine activity of DPC@NPs in vivo

The anti-inflammatory effect of DPC@NPs in early inflammation was then evaluated in vivo in the dental pulp tissues of Sprague-Dawley (SD) rats. Before this, the results of the cell counting kit-8 (CCK-8) experiment showed that various concentrations of DPC@NPs had no apparent adverse effect on the cell viability (Fig. S11) and cell proliferation (Fig. S12) of DPCs. Early pulpitis is characterized by inflammation localized to the coronal pulp, and it was selected as an early inflammation model.^31^ To determine the early pulpitis triggered by LPS in vivo, histologic observations were conducted at different time points (24, 48, and 72 h) (Fig. S13). In contrast with nontreated teeth, intense infiltration of the inflammatory cells was observed in the major part of the pulp when teeth were treated with *E. coli* LPS. Infiltrated cells were observed to be limited in the coronal pulp before 24 h, while the distribution of inflammatory cells progressed to the radicular canal (under the root canal orifice) at 48 and 72 h. These data revealed that experimentally induced early pulpitis was successfully established at 24 h.

**Fig. 6:**
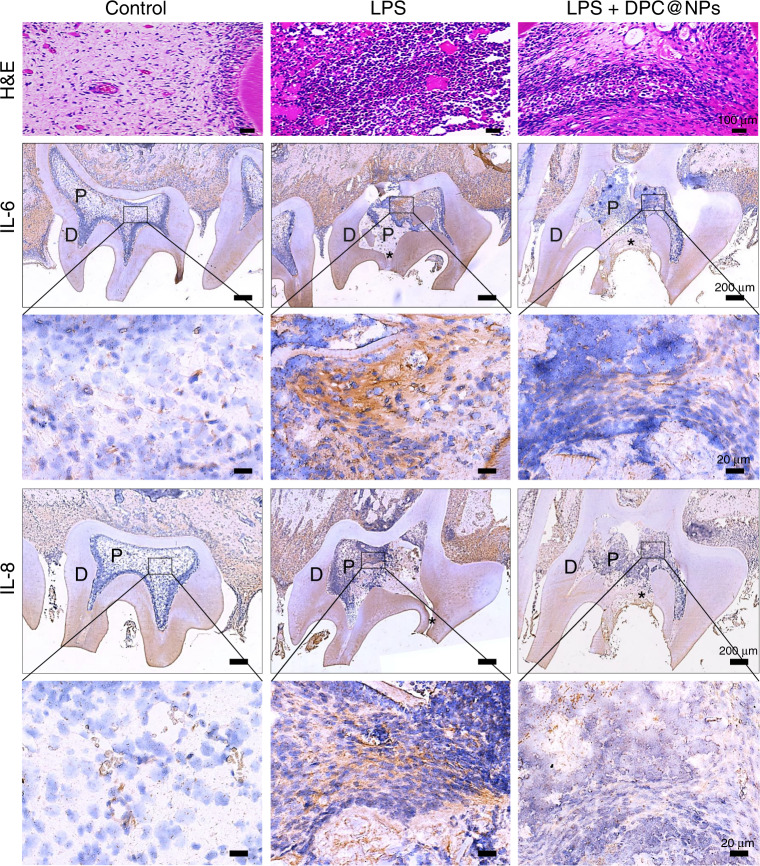
H&E staining and IHC analysis of IL-6 and IL-8 in the phrophylactic regimens. D indicates the dentin, and P indicates the pulp, while the asterisk represents the exposed area of the pulp

**Fig. 7:**
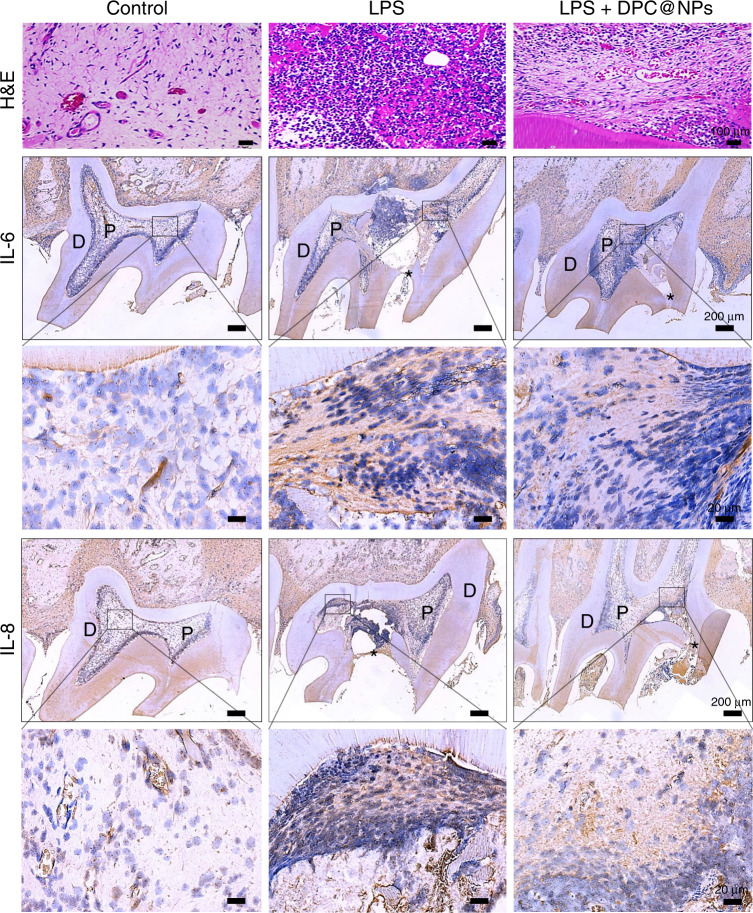
H&E staining and IHC analysis of IL-6 and IL-8 in the therapeutic regimens. D indicates the dentin, and P indicates the pulp, while the asterisk represents the exposed area of the pulp

Thereafter, pulp tissues with experimental early pulpitis were treated with rat DPC@NPs (rDPC@NPs), which were fabricated using rat DPCs (rDPCs) and PLGA in advance (Fig. S14–15). Then, the mixture of rDPC@NPs and *E. coli* LPS was injected into the pulp chamber as prophylactic regimens, or *E. coli* LPS was first administered for 1 h, followed by the injection of rDPC@NPs as therapeutic regimens.^30^ Hematoxylin and eosin (H&E) and immunohistochemistry (IHC) were performed to analyze the severity of the local inflammatory response after 24 h. H&E staining revealed more infiltration of inflammatory cells in the pulp tissues when they were stimulated by *E. coli* LPS than in the control group. However, the administration of rDPC@NPs dramatically alleviated the infiltration of inflammatory cells (Figs. 6–7). Consistent with the results of H&E staining, the IHC results showed that the expression of IL-6 and IL-8 was markedly upregulated in the *E. coli* LPS treatment group but not in the control group. This effect of *E. coli* LPS was significantly attenuated by rDPC@NPs both in the prophylactic (Fig. 6) and therapeutic regimens (Fig. 7).

### Anti-inflammatory mechanism of DPC@NPs in vitro and in vivo

**Fig. 8:**
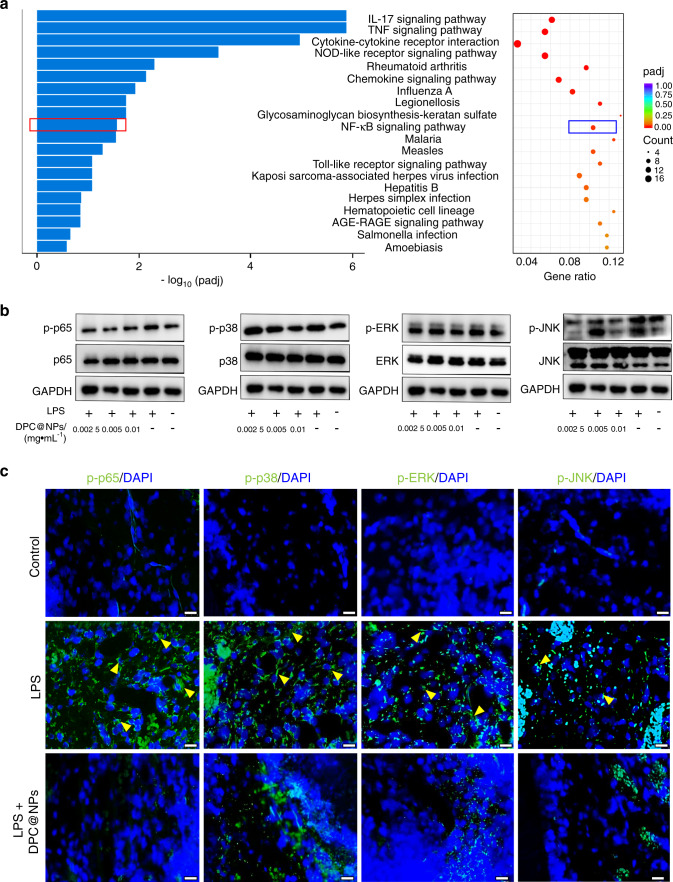
Effect of DPC@NPs on *E*. *coli* LPS-stimulated activation of signaling pathways. **a** Top 20 significantly enriched KEGG signaling pathway terms between the DPCs treated with *E. coli* LPS + DPC@NPs and *E. coli* LPS. **b** WB analysis of the effect of various concentrations of DPC@NPs on the expression of p-p65, p-p38, p-ERK, and p-JNK in vitro. **c** IF analysis of the effect of DPC@NPs on the expression of p-p65, p-p38, p-ERK, and p-JNK (indicated by yellow triangle) in vivo. bar = 20 μm

To investigate how DPC@NPs inhibit the expression of inflammatory cytokines, a Kyoto Encyclopedia of Genes and Genomes (KEGG) pathway enrichment analysis was conducted. Among the top 20 enriched KEGG pathways, the NF-κB signaling pathway was always a significantly altered pulpitis-related signaling pathway (Fig. 8a and Fig. S16). We next examined the effect of DPC@NPs on the NF-κB signaling pathway in vitro and in vivo. In addition, the p38 MAPK kinase signaling pathway was also examined. p65 and p38, ERK, JNK are major signaling components of NF-κB and p38 MAP kinase heterodimers, respectively. Phosphorylation of p65 (p-p65), and p38 (p-p38), ERK (p-ERK), JNK (p-JNK) plays an important regulatory role in inflammatory signal transduction.^32^ Therefore, we quantified the changes in the expression of these proteins along with their phosphorylation using densitometry. Our in vitro results were consistent with previous findings showing that *E. coli* LPS upregulated the expression of p-p65, p-p38, p-ERK, and p-JNK (Fig. 8b) compared with their expression levels in the control group.^33^ However, DPC@NPs suppressed the expression of these crucial signaling components (Fig. S17).

The results of immunofluorescence (IF) staining in vivo were consistent with those of WB in vitro. IF staining showed that low fluorescence was detected in the control group, while the fluorescence intensity increased after pulp tissues were treated with *E. coli* LPS for 24 h. However, the fluorescence signals of p-p65, p-p38, p-ERK, and p-JNK (Fig. 8c and Fig. S18) decreased upon the application of DPC@NPs.

## Discussion

Effective inflammation control poses a great challenge in many inflammatory diseases. Multitargeted treatment in the early stage is helpful in suppressing the severity of inflammation. Immune cell membrane-camouflaged nanoparticles have made tremendous progress in broad-spectrum anti-inflammatory treatment. In this study, engineered TLR4-presenting fibroblast membrane-camouflaged nanoparticles (DPC@NPs) were fabricated for early inflammation treatment. TLR4 is a model antigen available to mediate LPS recognition and neutralization and is rarely expressed on unstimulated DPCs.^28,34–36^ DPCs were engineered to display TLR4 antigens in an LPS stimulatory context during incubation.^22^ After vesicle extraction and cell membrane-nanoparticle assembly, a membrane shell with maintained TLR4 activity is extremely necessary. WB experiments validated the transfer of TLR4 from DPCs and vesicles of *E. coli* LPS-stimulated DPCs to DPC@NPs. DPCs contain not only membrane receptor proteins but also intracellular proteins. However, the vesicles of *E. coli* LPS-stimulated DPCs and DPC@NPs lacked intracellular proteins and contained only membrane proteins.^17^ When loading the same amount of proteins, the vesicles of the *E. coli* LPS-stimulated DPCs and DPC@NP groups matched well and had higher membrane receptor protein expression because their protein compositions were purer and the proportion of TLR4 was higher than that of DPCs.^17,24^ The retention and enrichment of the key surface antigen TLR4 on the surface of DPC@NPs (engineered TLR4-presenting fibroblast membrane-camouflaged nanoparticles) makes them capable of specific binding to LPS.^24^

Subsequently, we evaluated the functional neutralization of LPS by DPC@NPs. A GO analysis was conducted, and the results showed that the response to the LPS signaling pathway was the most enriched BP term when the DPCs were stimulated with LPS alone. With the addition of various concentrations of DPC@NPs, the enriched BP terms changed significantly. BP is essential to evaluating the LPS activity.^37^ Therefore, the BP results indicated that the biological activity of LPS was inhibited by the DPC@NPs. In addition, several crucial LPS-regulated genes in the response to the LPS signaling pathway were significantly activated in DPCs stimulated with *E. coli* LPS. However, the addition of DPC@NPs significantly downregulated these genes in DPCs. These results further demonstrated that DPC@NPs inhibited LPS-induced signaling pathway activation in DPCs.

To determine how DPC@NPs play an inhibitory role on *E. coli* LPS, the removal capacity of DPC@NPs was quantified.^24^ The binding studies confirmed that DPC@NPs at concentrations ranging from 0.002 5 to 0.01 mg·mL^−1^ had a powerful capability to functionally neutralize *E. coli* LPS. Meanwhile, DPC@NPs exhibited concentration-dependent binding to *E. coli* LPS.^24^ On this basis, DPCs were added to the system to explore the competitive binding ability between various concentrations of DPC@NPs and DPCs to LPS. In this part, we pay our attention to the dynamic changes of the binding capacity of DPC@NPs and DPCs to LPS with the concentration changes of DPC@NPs (0.01, 0.005, and 0.0025 mg·mL^−1^). We found that the higher concentration of DPC@NPs resulted in improved competitive binding capacity. According to the reported literature, coating membranes onto nanoparticles significantly increased the surface-to-volume ratio of the given membrane materials which may be favorable for LPS absorption.^24^ In addition, the maintained membrane protein TLR4 on the DPC@NPs would be conducive to functional neutralization of LPS.^22^ The improved neutralization capability of higher concentrations of DPC@NPs is likely attributable to more key surface protein TLR4 being involved in the specific LPS-binding process.^30^ Therefore, preserved membrane activities were successfully applied for effective LPS neutralization by DPC@NPs. Collectively, our fibroblast membrane-camouflaged nanoparticles showed great potential to be used for sustained LPS neutralization in the inflammation process.

According to the results of DPC@NPs absorbing *E. coli* LPS, DPC@NPs outside the cells may sequester bacterial virulence, and block DPCs activation to achieve multitargeted anti-inflammatory treatment.^19,24^ Therefore, we studied the anti-inflammatory cytokine activity of DPC@NPs using an RNA-Seq analysis. The results indicated that the various concentrations of DPC@NPs inhibited gene expression closely related to inflammation in DPCs stimulated by *E. coli* LPS. For a wide range of inflammatory cytokines playing different roles in the course of inflammation, multitargeted inhibition of cytokines helps to achieve a stronger suppressive effect in the pathogenesis of inflammation compared with the existing anti-inflammatory approaches, which can only inhibit one or a few inflammatory molecules.^19^

Among a plethora of inflammatory cytokines involved in the inflammatory process, IL-6 and IL-8 have been considered to be two of the most important regulatory molecules in the inflammatory process and act as major mediators of the host response in pulpitis.^38–40^ They can be generally induced by many bacterial antigens, such as LPS, and are responsible for recruiting other inflammatory cells to the pulp chamber. Their overexpression may exacerbate the inflammatory response.^41,42^ Therefore, IL-6 and IL-8 were selected as representative inflammatory cytokines to reflect the influence of DPC@NPs on the severity of pulpal inflammation in vitro and in vivo. These results demonstrated the effective protection of DPC@NPs against the production of IL-6 and IL-8 in the early stage of inflammation both in the prophylactic and therapeutic regimens. DPCs form the majority of cells in dental pulp tissues and play a significant role in the pathogenesis of pulpitis. DPCs can bind to LPS and produce a variety of inflammatory cytokines, including IL-6 and IL-8, which mediate progressive inflammation in pulp tissues.^10^ Notably, DPC@NPs inherited the function of natural DPCs, which can specifically absorb and neutralize LPS. The competitive biological binding with LPS between DPC@NPs and normal DPCs lowered the concentration of LPS in pulp tissues.^30^ More DPCs were free from LPS stimulation, leading to decreased inflammation in the early stage. As the inflammatory response often progresses locally to systemically in vivo, the inhibition of local inflammatory cytokine expression in the early stage will largely avoid systemic disorders.^3,43,44^ Similar to DPCs in dental pulp tissues, other resident fibroblasts in various inflammatory diseases are capable of detecting bacterial virulence and mounting the inflammatory response.^45^ Therefore, fibroblast membrane-camouflaged nanoparticles are capable of providing protection for resident cells against offending virulences in the early stage of inflammation.

**Fig. 9:**
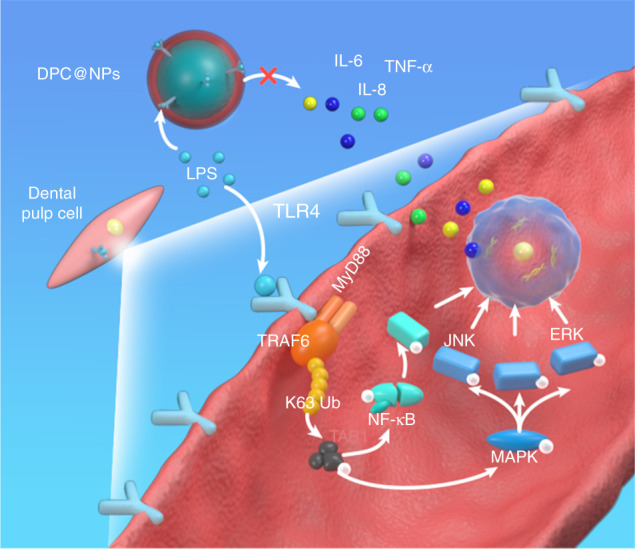
Schematic illustration depicting the suppressive mechanism of DPC@NPs on signaling pathways in DPCs. DPC@NPs act as an ideal decoy of DPC-targeted LPS, preventing the LPS-stimulated intracellular NF-κB and p38 MAPK signaling pathways from activating. The inhibition of the activation of signaling pathways lessens the production of multiple inflammatory cytokines

The production of inflammatory cytokines has been proven to be introduced by intricate intracellular signaling pathways. The KEGG analysis indicated that the NF-κB signaling pathway may be closely associated with the inhibitory effect of DPC@NPs on pulpitis. In addition, the p38 MAP kinase signaling pathway has been reported to act as an upstream or downstream signaling pathway of NF-κB to play an essential role in the introduction of inflammatory cytokines in pulpitis.^46^ Therefore, the NF-κB and p38 MAPK kinase signaling pathways were both examined in vitro and in vivo, and they indicated lower levels of NF-κB and p38 MAP kinase signaling pathway activation by DPC@NPs. LPS is thought to promote the expression of cytokines by binding to TLR4 on DPCs, which then activates myeloid differentiation primary response gene 88 (MyD88).^47^ MyD88 sequentially recruits and activates subsequent molecules, including IL-1R-associated kinase-4 (IRAK4), IRAK1, and TNF-receptor-associated factor-6 (TRAF6).^48^ Then, the NF-κB and p38 MAP kinase signaling pathways are activated. Thus, the TLR4/MyD88-mediated NF-κB and p38 MAP kinase pathways are considered to be crucial signaling axes in LPS-induced pulpitis (Fig. 9). Collectively, the inhibitory effect of DPC@NPs on inflammatory cytokines seems to be related to the NF-κB and p38 MAP kinase signaling pathways. According to the inflammation regulation mechanism of DPC@NPs in pulpitis, fibroblast membrane-camouflaged nanoparticles exert an anti-inflammatory role by intervening in LPS-activated TLR4-related signaling pathways.

## Conclusions

In this work, we successfully developed biomimetic fibroblast membrane-camouflaged nanoparticles (DPC@NPs) that inherited the membrane function of natural fibroblasts. From multiple perspectives, including materials science, bioinformatics, and molecular biology, fibroblast membrane-camouflaged nanoparticles have been demonstrated to act as sentinels to compete with fibroblasts to absorb invading virulences, which dampens the inflammatory cascade in the early stage at the macro level and micro molecular level in vitro and in vivo. Our work demonstrates the considerable potential of fibroblast membrane-camouflaged nanoparticles for the management of inflammation, especially in the early stage. Meanwhile, it also provides insights needed to widen the sophisticated applications employing plasma membranes from natural cells for the treatment of inflammatory diseases.

## Materials and methods

### Culture and characterization of DPCs

All relevant experiments were approved by the Institutional Review Board of West China Hospital of Stomatology, Sichuan University (WCHSIRB-D-2021-082). Human dental pulp tissues were separated from caries-free premolars extracted for orthodontic purposes.^49^ Then, pulp tissues were cut and the shredded tissues were placed in a solution of 3 mg·mL^−1^ collagenase type I (Sigma, USA) at 37 °C for 20 min. Loose pulp tissues were softly transferred to a culture vessel (Corning, USA) and covered with Dulbecco’s modified Eagle medium (DMEM, Gibco, USA) containing 20% fetal bovine serum (Gibco, USA) and 1% penicillin-streptomycin (100 UmL^–1^, Gibco). Human dental pulp cells (hDPCs) were cultured in an incubator under 5% CO_2_ at 37 °C, and passages 3–6 were used in the subsequent experiments.

The third passage of hDPCs was characterized by immunocytochemical staining.^49,50^ Briefly, hDPCs were fixed in 4% paraformaldehyde (Biosharp, China) at room temperature for 20 min. Then, hDPCs were incubated with hydrogen peroxide for 10 min to block endogenous peroxidase activity. After washing twice with 1 × phosphate buffered solution (PBS, Gibco, USA), hDPCs were incubated with bovine serum albumin (Absin, China) at room temperature for 5 min and then with primary antibodies (anti-vimentin antibody, Absin, 1:1 000, China; anti-pankeratin antibody, Cell Signaling Technology, 1:1 000, USA) at 37 °C for 20 min.^49^ Then, hDPCs were rinsed three times and incubated with horseradish peroxidase-conjugated secondary antibody (Absin, China) at room temperature for 10 min. Finally, the color was developed with 3,3′-diaminobenzidine (DAB, Absin, China) solution at room temperature for 5 min. After nuclear staining with hematoxylin staining solution (Biosharp, China), the cells were imaged using a bright-field microscope (Nikon, E200, Japan).

### DPC activation assay

qRT–PCR was performed to detect the gene expression level of *TLR4* after DPCs were stimulated with different concentrations of *E. coli* LPS (*E. coli O55:B5*, Sigma, 10 μg·mL^−1^ and 1 μg·mL^−1^, respectively) at different time points (1, 3, 6, 12, and 24 h).^22,51^ The cells were then washed 3 times and collected. Total mRNA in DPCs was extracted using TRIzol (Takara, Japan), followed by reverse transcription using the PrimeScript RT reagent kit (Takara, Japan). After mRNA was reverse transcribed to complementary DNA (cDNA), the cDNA levels were measured by qRT–PCR using the SYBR Premix DimerEraser kit (Takara, Japan). All experimental steps were performed in accordance with the manufacturer’s manuals. Each sample was analyzed 3 times, and the mean value of 2^-ΔΔCt^ was calculated. Compared with the control group, which was set to 1, fold changes of mRNA expression in the experimental groups were described. The primers for *TLR4* used in this study were as follows: forward, 5′-CTG CAA TGG ATC AAG GAC CA-3′, and reverse, 5′-TTA TCT GAA GGT GTT GCA CAT TCC-3′. Glyceraldehyde-3-phosphate dehydrogenase (*GAPDH*) was used as an internal control, and its primers were as follows: forward, 5′-ATG GGG AAG GTG AAG GTC GGA GTC-3′, and reverse, 5′-GCT GAT GAT CTT GAG GCT GTT GTC-3′.

Flow cytometry of TLR4 expression on the membrane surface of DPCs was further used to confirm the qRT–PCR results at the protein level. The DPCs in the control group and the DPCs with the highest TLR4 expression in the qRT–PCR were collected. Cells were stained with rabbit anti-TLR4 (Bioss, China) and FITC-conjugated goat anti-rabbit IgG (Proteintech, China). The TLR4 receptor on the DPCS was then calculated by flow cytometer, and data analysis was performed via FlowJo.

### Preparation of membrane-derived vesicles from activated DPCs

The plasma membrane of DPCs was harvested by the chemical reagent method followed by high-speed centrifugation.^52^ Briefly, DPCs were seeded into T75 flasks and cultured for 48–72 h. When approximately 90% confluent, the DPCs were stimulated with *E. coli* LPS (10 μg·mL^−1^) at 37 °C for 6 h. The cells were obtained and suspended in Membrane and Cytosol Protein Extraction Kit A (Beyotime, China) containing 1% phenylmethanesulfonyl fluoride (PMSF, 10 mmol·L^–1^, Beyotime, China) and cooled in an ice bath for 20 min. Then, DPCs were alternately frozen and thawed 5 times. The solution was then centrifuged at 12 000 r·min^–1^ at 4 °C for 1 h. The supernatant was discarded, and the precipitate was collected. Then, the membrane precipitate was extruded through a 200 nm polycarbonate membrane using a mini extruder (Avestin, LF-1, Canada) to obtain membrane-derived vesicles. To verify the vesicles, they were stained with the cell membrane probe DiO (10 μmol·L^–1^, Beyotime, China). In addition, the morphology of DPC membrane-derived vesicles was visualized under TEM (JEOL, JEM-1400 Plus, Japan). The resultant DPC membrane-derived vesicles were stored in 1 × PBS at 4 °C until use.^53^

### Synthesis of PLGA nanoparticles

PLGA nanoparticles were prepared via a nanoprecipitation process.^54^ To synthesize nanoparticle cores, 10 mg PLGA (50:50, Lactel Absorbable Polymers, USA) was dissolved in 1 mL N,N-dimethylformamide. Then, 200 μL PLGA (10 mg·mL^−1^) was added dropwise into 20 mL deionized H_2_O and stirred for 2 min. The solution was placed in a dialysis bag (1 000 kD) on a magnetic stirrer overnight. For the fluorescence imaging experiments, DiD (5 mM, Beyotime, China) was encapsulated in PLGA cores (0.1 wt%). The morphology of the PLGA nanoparticles was visualized using TEM (JEOL, JEM-1400 Plus, Japan).

### Fusion of DPC membrane-derived vesicles with PLGA nanoparticles

DPC@NPs were obtained through mechanical extrusion.^16,55^ Prepared DPC membrane-derived vesicles were resuspended in 300 μL of 1 × PBS and mixed with 300 μL PLGA (0.1 mg·mL^−1^). The mixture was then sonicated with a sonicator bath (FS30D, 42 kHz, 100 W) for 2 min and repeatedly coextruded through a 200 nm polycarbonate membrane using a mini extruder (Avestin, LF-1, Canada) 30 times. The DPC@NPs were serially diluted with DMEM, and the final concentrations of DPC@NPs were 0.01, 0.005, and 0.002 5 mg·mL^−1^.

### Characterization of DPC@NPs

The morphology of DPC@NPs was examined to confirm the PLGA-membrane association. DPC@NPs were negatively stained with phosphotungstic acid (1%) and visualized using TEM (JEOL, JEM-1400 Plus, Japan).^56^ The size and zeta potential of PLGA nanoparticles, DPC vesicles and DPC@NPs were measured using a dynamic light-scattering instrument (Malvern, Zetasizer Nano ZS, UK) and a zeta potential analyzer (HORIBA, SZ-100, Japan), respectively.^17^ The Tyndall effect was used to verify the optical phenomenon of colloidal membrane-camouflaged nanoparticles.^21^ The optical phenomenon was tested in a dark room, and the red laser beam passed through the solution with and without DPC@NPs simultaneously. Subsequently, the stability of DPC@NPs was assessed in 1× PBS at room temperature over a span of 28 days, and their size and PDI were monitored using a dynamic light-scattering instrument (Malvern, Zetasizer Nano ZS, UK).^30^

### Characterization of the integrity of DPC@NPs

To verify the colocalization of DPC membrane-derived vesicles and PLGA nanoparticles, they were labeled with DiO and DiD, respectively.^54^ Then, RAW 264.7 cells were cultured and incubated with dual-fluorophore-labeled DPC@NPs at 37 °C for 6 h. After washing and fixing with tissue fixative, the nuclei of RAW 264.7 cells were stained with 4′,6-diamidino-2-phenylindole (DAPI, 10 μg·mL^−1^, Solarbio, China) at room temperature for 5 min. The resulting fluorescent images were obtained using a fluorescence microscope (OLYMPUS, IX73, Japan) and a confocal laser scanning microscope (CLSM, OLYMPUS, FV1000, Japan). Fluorescence signals of DAPI (blue), DiD (red), and DiO (green) were acquired under DAPI, CY5, and FITC filters.

### Characterization of the key membrane receptor (TLR4)

The protein contents of DPC@NPs were characterized by SDS–PAGE.^57^ Membrane proteins of DPCs, vesicles of *E. coli* LPS-stimulated DPCs, and DPC@NPs were extracted using a total protein extraction kit radioimmunoprecipitation assay (RIPA, Beyotime, China) containing 1% PMSF. Then, these lysis solutions were centrifuged at 12 000 r·min^–1^ at 4 °C for 15 min. The supernatant was carefully transferred to a clean microfuge tube and the protein concentration was measured using the BCA protein assay kit (Beyotime, China). After the denaturation of the protein, the total proteins were separated by 10% free staining gel (Bio–Rad, USA) in the running buffer at 90 V for 1.5 h. Subsequently, WB was used to confirm the presence of the characteristic receptor TLR4.^58^ Total membrane proteins were transferred onto a polyvinylidene difluoride (PVDF) membrane (0.22 μm, Millipore, USA) at 200 mA for 2 h. PVDF membranes were blocked with 5% skim milk (Biofroxx, Germany) for 1 h. Then, they were incubated with the primary antibody against TLR4 (mouse anti-TLR4 antibody, Proteintech, 1:1 000, China) at 4 °C for 12 h and then with the HRP-conjugated goat anti-mouse secondary antibody (Signalway Antibody, 1:5 000, USA) at room temperature for 1 h. Finally, the detection reagent Super ECL Plus (US EVERBRIGHT, China) was dipped onto PVDF membranes, and bands of proteins were observed using the BIO-RAD Gel Doc XR + imaging system.

### RNA-seq analysis

RNA samples were collected from Group 1: control group; Group 2: DPCs stimulated with 10 μg·mL^−1^ *E. coli* LPS; Group 3: DPCs stimulated with 10 μg·mL^−1^ *E. coli* LPS and 0.01 mg·mL^−1^ DPC@NPs; Group 4: DPCs stimulated with 10 μg·mL^−1^ *E. coli* LPS and 0.005 mg·mL^−1^ DPC@NPs; and Group 5: DPCs stimulated with 10 μg·mL^−1^ *E. coli* LPS and 0.002 5 mg·mL^−1^ DPC@NPs. Samples were sent to the Novogene Institution for mRNA enrichment, cDNA synthesis, library construction, and sequencing with the Illumina platform.^59^ Clean reads for subsequent analysis were obtained after filtering the original data, checking the error rate of sequencing, and distributing the GC content. Then, the clean reads were precisely compared with the reference gene sequence using HISAT2 software. According to the acquired gene alignment on the reference gene sequence, the gene expression levels were counted using Subread software. The LPS signaling-related genes in each group were integrated to create the heatmap. The total altered expression genes in each group were displayed by a volcano plot and the heatmap, while the differentially expressed genes related to inflammation were shown in the heatmap. In addition, a KEGG analysis was used to observe the changes in related intracellular signaling pathway enrichment.

### Examination of the binding between DPC@NPs and *E. coli* LPS

The binding ability of DPC@NPs was examined following a previous report.^24^ Briefly, serial dilutions of DPC@NPs ranging from 0.01 to 0.002 5 mg·mL^−1^ were mixed with *E. coli* LPS (10 μg·mL^−1^) and incubated at 37 °C for 30 min. Then, the mixture was collected and centrifuged at 12 000 r·min^–1^ for 15 min. The remaining *E. coli* LPS in the supernatant was measured using an *E. coli* LPS enzyme-linked immunosorbent assay (ELISA) kit (MEIMIAN, China).

Then, the competitive binding capacity among various concentrations of DPC@NPs (0.01, 0.005, and 0.002 5 mg·mL^−1^) was further examined. Confluent DPCs in each well of six-well plates were stimulated with an equal volume of FITC (excitation = 490 nm/emission = 520 nm)-*E. coli* LPS conjugate (Sigma, USA) followed by the administration of different concentrations of DPC@NPs. These mixtures were incubated at 37 °C for 1 h. FITC-*E. coli* LPS intensities that were pelleted into supernatant or combined with DPCs on the bottom of 6-well plates were compared among various concentrations of DPC@NPs.

To evaluate the short-term binding stability, 0.01 mg·mL^−1^ DPC@NPs were mixed with FITC-*E. coli* LPS conjugate. Following incubation and centrifugation, the fluorescence intensity of FITC in the supernatant was measured. Then, after storage of DPC@NPs for 24 and 72 h, the ability of DPC@NPs to neutralize *E. coli* LPS was further evaluated.

### qRT–PCR analysis

The prophylactic and therapeutic efficacies of DPC@NPs were examined. DPCs were stimulated with 1 mL *E. coli* LPS (10 μg·mL^−1^), and 1 mL DPC@NP solution at various dosages (0.01, 0.005, and 0.002 5 mg·mL^−1^) was added at the same time as the prophylactic regimens.^33^ On the other hand, 1 mL DPC@NP solution at various concentrations was added after the DPCs were stimulated with *E. coli* LPS for 1 h as the therapeutic regimen. DPCs were collected at 12 and 24 h to evaluate the mRNA expression of *IL-6* and *IL-8*. The primer sets were as follows: (1) *IL-6*: forward, 5′-ACT CAC CTC TTC AGA ACG AAT TC-3′, and reverse, 5′-CCA TCT TTG GAA GGT TCA GGT TG-3′; (2) *IL-8*: forward, 5′-CTG GCC GTG GCT CTC TTG-3′, and reverse, 5′-CCT TGG CAA AAC TGC ACC TT-3′; (3) *GAPDH*: forward, 5′-ATG GGG AAG GTG AAG GTC GGA GTC-3′, and reverse, 5′-GCT GAT GAT CTT GAG GCT GTT GTC-3′. The threshold cycle number in qRT–PCR was 40.

### ELISA

To measure the concentrations of the inflammatory cytokines secreted into the supernatant, the culture medium of each well was collected and frozen at −20 °C for analysis. The concentrations of IL-6 and IL-8 in the medium were determined following the manufacturer’s instructions (MEIMIAN, China). The color change (blue to yellow) after the addition of the stop solution was monitored by measuring the absorbance at 450 nm. According to the absorbance value, a standard curve was constructed. The expression levels of IL-6 and IL-8 in the lysate were normalized to the protein concentration.

### CCK-8 assay

DPCs were seeded into a 96-well plate at a density of 4 × 10^3^ cells per well. Then, serial concentrations of DPC@NPs (0.01, 0.005, and 0.002 5 mg·mL^−1^) were added to the wells. After incubation at 37 °C for 24, 48, and 72 h, the CCK-8 (ApexBio, USA) assay was performed to evaluate the cell viability and proliferation rate of DPCs.^60^ According to the manufacturer’s instructions, the optical density (OD) value of each well was determined at a wavelength of 450 nm using a microplate reader (Thermo Fisher, USA).

### Experimentally induced early pulpitis in rats

SD rats (7 weeks, male, Dashuo, China) weighing 220–280 g were fed a standard laboratory rat diet and allowed free access to water. After adaptive feeding for one week, 12 SD rats were taken and randomly divided into four groups (3 rats per group). The rats were anesthetized with sodium pentobarbital. Following this, the left upper first molar was occlusally exposed using 1/4 round burs.^61^ *E. coli* LPS solution was applied to the pulp with a 20 μL microinjector. The cavity was sealed with glass ionomer cement (GC Fuji IX, Japan). Untreated normal teeth were used as the control group.^29^ The SD rats were sacrificed at 24, 48, and 72 h after the administration of *E. coli* LPS.^29^ After the extraction of the maxillary bones, the tooth was fixed in 4% paraformaldehyde fix solution, decalcified in 0.5 M EDTA for 45 d, embedded in paraffin, and sectioned to 5 μm thickness. The slices were stained with H&E.^62^

### Effects of DPC@NPs on inflammatory cell recruitment and inflammatory cytokine production in vivo

The methods of cell culture and characterization of rDPCs, preparation of rDPC membrane-derived vesicles, and synthesis of rDPC@NPs were consistent with those of human DPC@NPs. Forty male SD rats weighing 220–280 g were used. In vivo experiments were performed by the simultaneous addition of *E. coli* LPS and DPC@NPs (*n* = 20) or the subsequent addition of DPC@NPs (*n* = 20). Similar to the operation of experimentally induced rat early pulpitis, the pulp of the left maxillary first molar was exposed to the depth of the diameter of the bur. Then, the mixture of *E. coli* LPS and DPC@NPs was injected into the pulp chamber through the exposed area as the prophylactic regimen. Molars without treatment were assigned as the control group. In the therapeutic regimen, *E. coli* LPS was added to the pulp chamber 1 h before DPC@NPs were administered. The cavity was sealed, and the experimental animals were allowed free access to food and water.

After the rats were sacrificed 24 h later, the maxillary bones were removed and fixed in 4% paraformaldehyde fix solution for 3 days. These samples were then decalcified in 0.5 mol·L^–1^ EDTA for 45 d and embedded in paraffin. The target teeth were sectioned serially at 5 μm in the mesiodistal plane. Then, these sections were stained with H&E or analyzed via IHC.^63^ In brief, paraffin sections were deparaffinized and incubated with 3% H_2_O_2_ at 37 °C for 10 min to block the activity of endogenous peroxidase.^64^ Then, the slides were placed in antigen-repairing solutions in an antigen repair box. After antigen retrieval, these sections were separately incubated with the following primary antibodies at 4 °C for 12 h: mouse anti-IL-6 antibody (Abcam, 1:200, UK) and rabbit anti-IL-8 antibody (Proteintech, 1:200, China). After incubation, the slices were incubated with a biotin-labeled secondary antibody (ZSGB-BIO, China) at 37 °C for 1 h. These slices were then washed three times and visualized using DAB color substrate solution. The nuclei were counterstained with hematoxylin for 5 min and differentiated with hydrochloric acid alcohol for 5 s. Following the dehydration step, the slices were sealed with resin. Images of each stained slice were captured using a light microscope (Motic, BA400, China) at magnifications of ×40 and ×400. The visual fields, which are located at the anterior of the inflammatory pulp tissues, were selected.

### WB

After extracting the protein with RIPA containing 1% protease inhibitor and phosphatase inhibitor (Beyotime, China), the sample lysates were subjected to SDS–PAGE and the proteins on the gel were transferred to PVDF membranes. The membranes were incubated with the following primary antibodies at 4 °C for 12 h: rabbit anti-p65 (Cell Signaling Technology, 1:1 000, USA), rabbit anti-p-p65 (Cell Signaling Technology, 1:1 000, USA), rabbit anti-p38 (Cell Signaling Technology, 1:1 000, USA), rabbit anti-p-p38 (Cell Signaling Technology, 1:1 000, USA), rabbit anti-ERK (Cell Signaling Technology, 1:1 000, USA), rabbit anti-p-ERK (Cell Signaling Technology, 1:1 000, USA), rabbit anti-JNK (Abcam, 1:1 000, UK), rabbit anti-p-JNK (Cell Signaling Technology, 1:1 000, USA), and rabbit anti-GAPDH (Cell Signaling Technology, 1:1 000, USA). HRP-conjugated goat anti-rabbit secondary antibody (Signalway Antibody, 1:5 000, USA) was combined with the primary antibody and allowed to react with the substrate. The pixel densities of the protein bands were measured with ImageJ software (v1.8.0, USA).

### IF

The mechanism of DPC@NPs on the production of inflammatory cytokines was pursued with further IF studies in vivo. For IF staining, deparaffinized slices were incubated with antibodies against rabbit p-p65 (Cell Signaling Technology, 1:1 600, USA), p-p38 (Cell Signaling Technology, 1:1 600, USA), p-ERK (Cell Signaling Technology, 1:200, USA), and p-JNK (Abcam, 1:50, UK) at 4 °C for 12 h. Then, the slices were incubated with FITC-conjugated goat anti-rabbit secondary antibody (Servicebio, 1:50, China) for 1 h, followed by staining of cell nuclei with DAPI. Fluorescent images were obtained using DAPI and FITC filters, and each image was captured at magnifications of ×50 and ×500.

### Statistical analysis

Statistical differences were analyzed by one-way ANOVA using GraphPad Prism 8 (GraphPad Software Inc., CA, USA). All the data are expressed as the mean ± standard. Probabilities as * and *n.s*. are marked in each figure. The asterisk indicates significance (*P* < 0.05), and n.s. represents no significance.

## Supplementary information

Supplemental Materials
