# Supplementary material for: Fibroblast membrane-camouflaged nanoparticles for inflammation treatment in the early stage
Source: Int J Oral Sci. 2021 Nov 16;13:39. doi: 10.1038/s41368-021-00144-2 (PMC8595357; doi:10.1038/s41368-021-00144-2)
Supplement: Supplementary file 1 — Supplemental Materials [file 41368_2021_144_MOESM1_ESM.docx]

## Fibroblast membrane-camouflaged nanoparticles for inflammation treatment in the early stage

Lizhong Sun^1,^ ^#^, Libang He^1,^ ^#^, Wei Wu^2^, Li Luo^2^, Mingyue Han^1^, Yifang Liu^1^, Shijie Shi^1^, Kaijing Zhong^1^, Jiaojiao Yang^1, *^, Jiyao Li^1, *^

**Affiliations:**

^1^ State Key Laboratory of Oral Diseases & National Clinical Research Center for Oral Diseases & Department of Cariology and Endodontics, West China Hospital of Stomatology, Sichuan University, Chengdu, China.

^2^ Key Laboratory for Biorheological Science and Technology of Ministry of Education, State and Local Joint Engineering Laboratory for Vascular Implants, Bioengineering College of Chongqing University, Chongqing, China.

**^#^ These authors contributed equally to the work**

*** Corresponding authors**

E-mail addresses: Jiaojiao Yang, email: [jiaojiao.yang@scu.edu.cn](mailto:jiaojiao.yang@scu.edu.cn); Jiyao Li, email: [jiyaoliscu@163.com](mailto:jiyaoliscu@163.com)


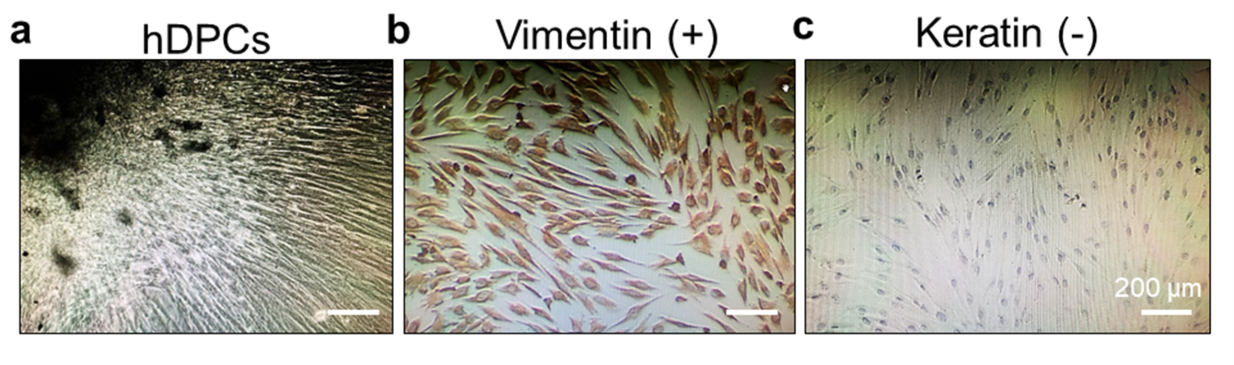


**Fig. S1** **a** Morphology of hDPCs. hDPCs were stained with **b** vimentin and **c** keratin by ICC.


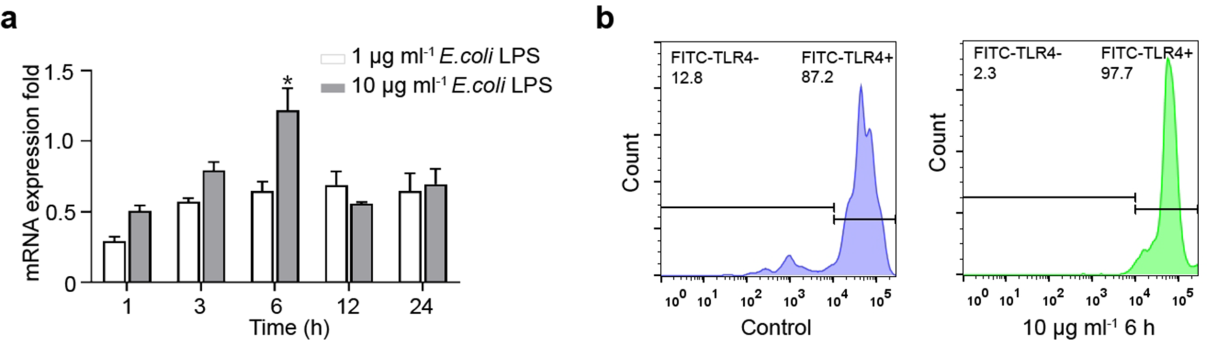


**Fig. S2** **a** The gene expression of *TLR4* in hDPCs stimulated with different concentrations of *E. coli* LPS (1 μg·mL^-1^ and 10 μg·mL^-1^) for different time (1, 3, 6, 12, and 24 h). Statistical analyses were performed by One-way ANOVA (** P< 0.05).* Data presented as mean ± s.d. (n = 3). **b** Flow cytometry of TLR4 expressions in hDPCs with and without stimulation of 10 μg·mL^-1^ LPS at 6 h.


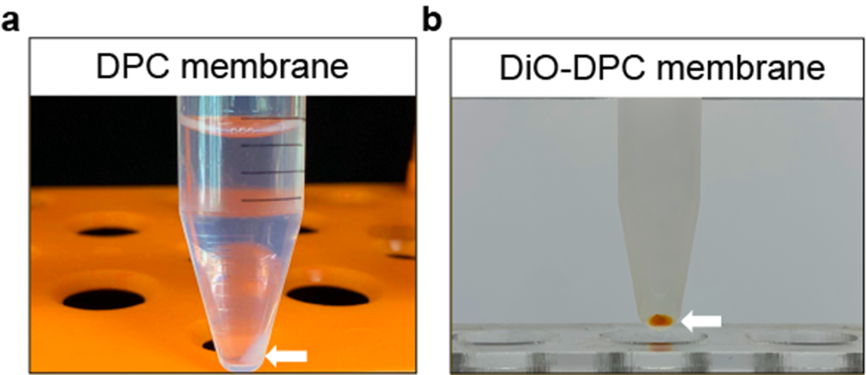


**Fig. S3** Representative optical images of **a** DPC vesicles and **b** DPC vesicles stained with DiO (orange), as indicated by arrows.


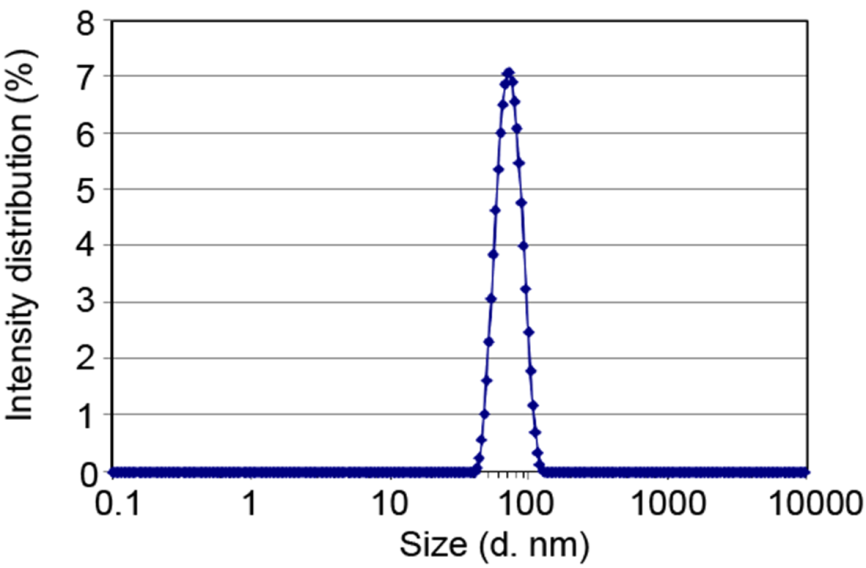


**Fig. S4** The hydrodynamic size of PLGA nanoparticles measured from DLS.


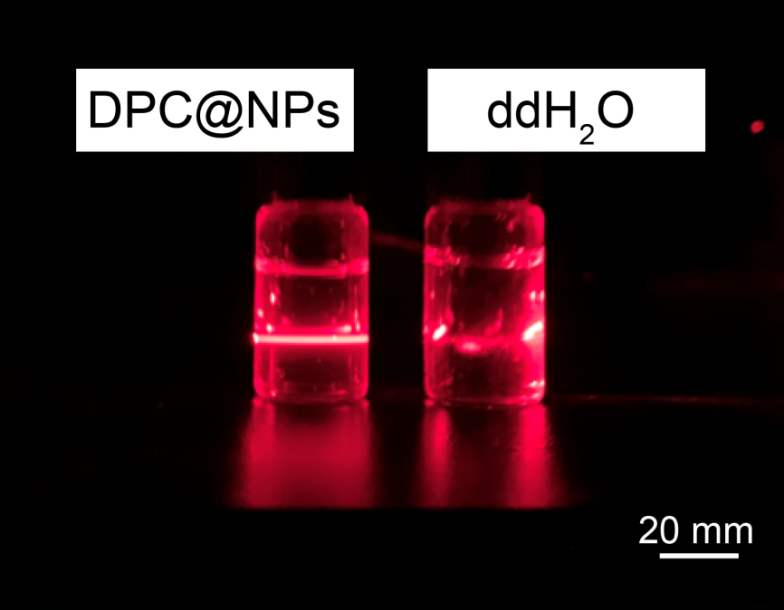


**Fig. S5** Photographs of the Tyndall effect of DPC@NPs (left) and ddH_2_O (right).


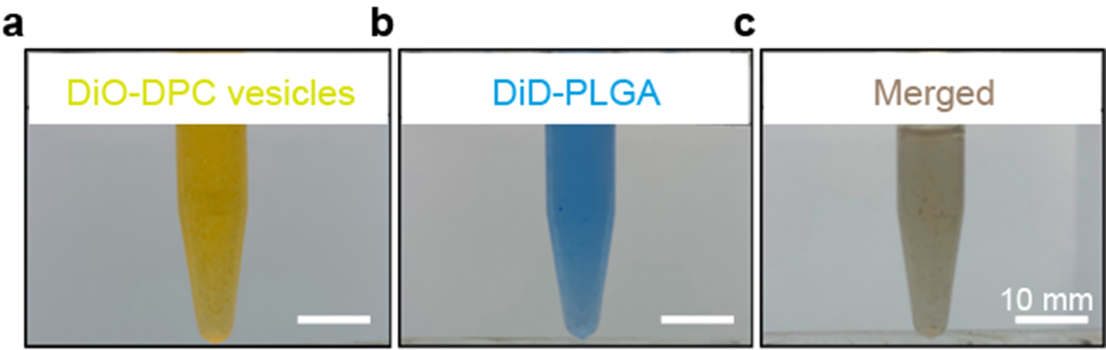


**Fig. S6** Photographs of **a** DiO-labeled DPC vesicles (yellow), **b** DiD-labeled PLGA (blue), and **c** DPC@NPs (grey).


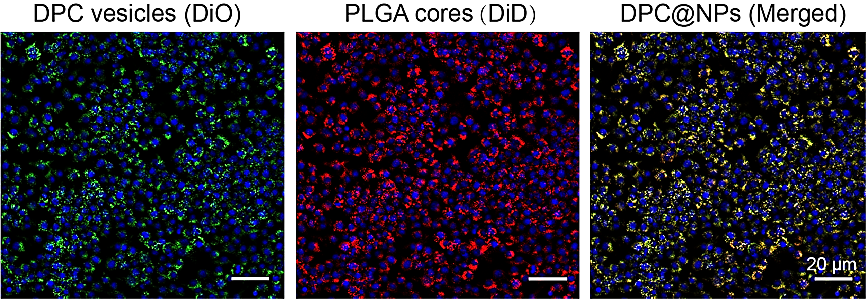


**Fig. S7** Representative fluorescent microscope images of the DPC membrane-derived vesicles (visualized with green DiO dyes), PLGA (visualized with red DiD dyes), and co-localization after nanoparticles endocytosis by RAW 264.7 (nuclei of RAW 264.7 were visualized with blue DAPI).


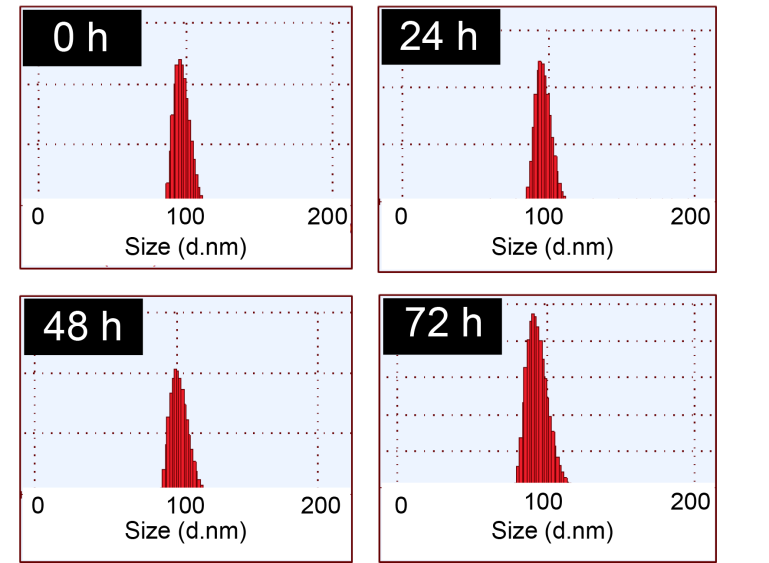


**Fig. S8** Short-term stability of the size distribution of DPC@NPs in 1 × PBS for 0, 24, 48, and 72 h measured from DLS.


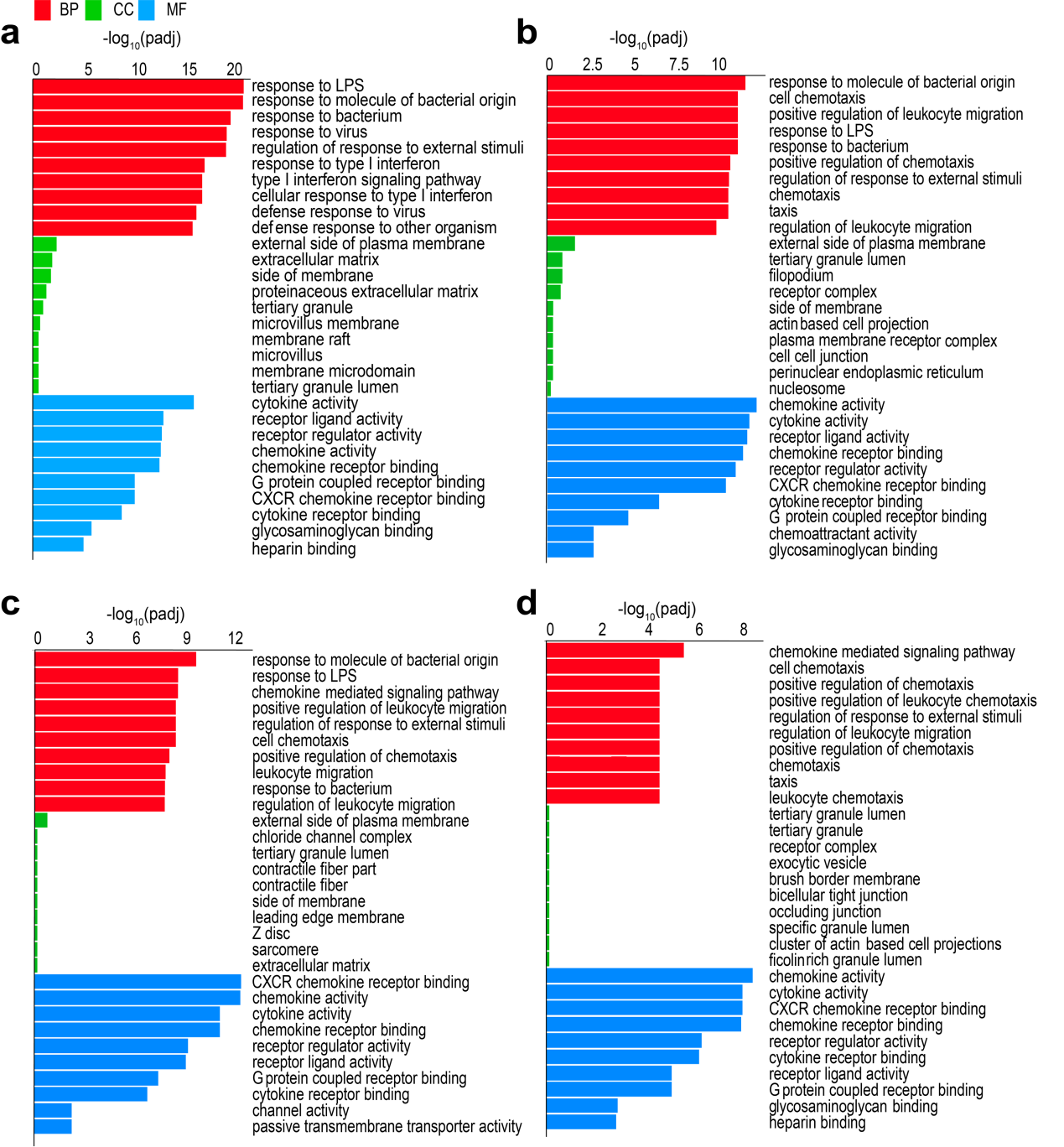
**Fig. S9** GO enrichment analysis including biological process (BP), cellular components (CC), and molecular functions (MF). The top 10 significantly enriched GO terms BP, CC, and MF between **a** control group and DPCs stimulated with LPS, **b** LPS and LPS + 0.01 mg·mL^-1^ DPC@NPs, **c** LPS and LPS + 0.005 mg·mL^-1^ DPC@NPs, and **d** LPS and LPS + 0.002 5 mg·mL^-1^ DPC@NPs.


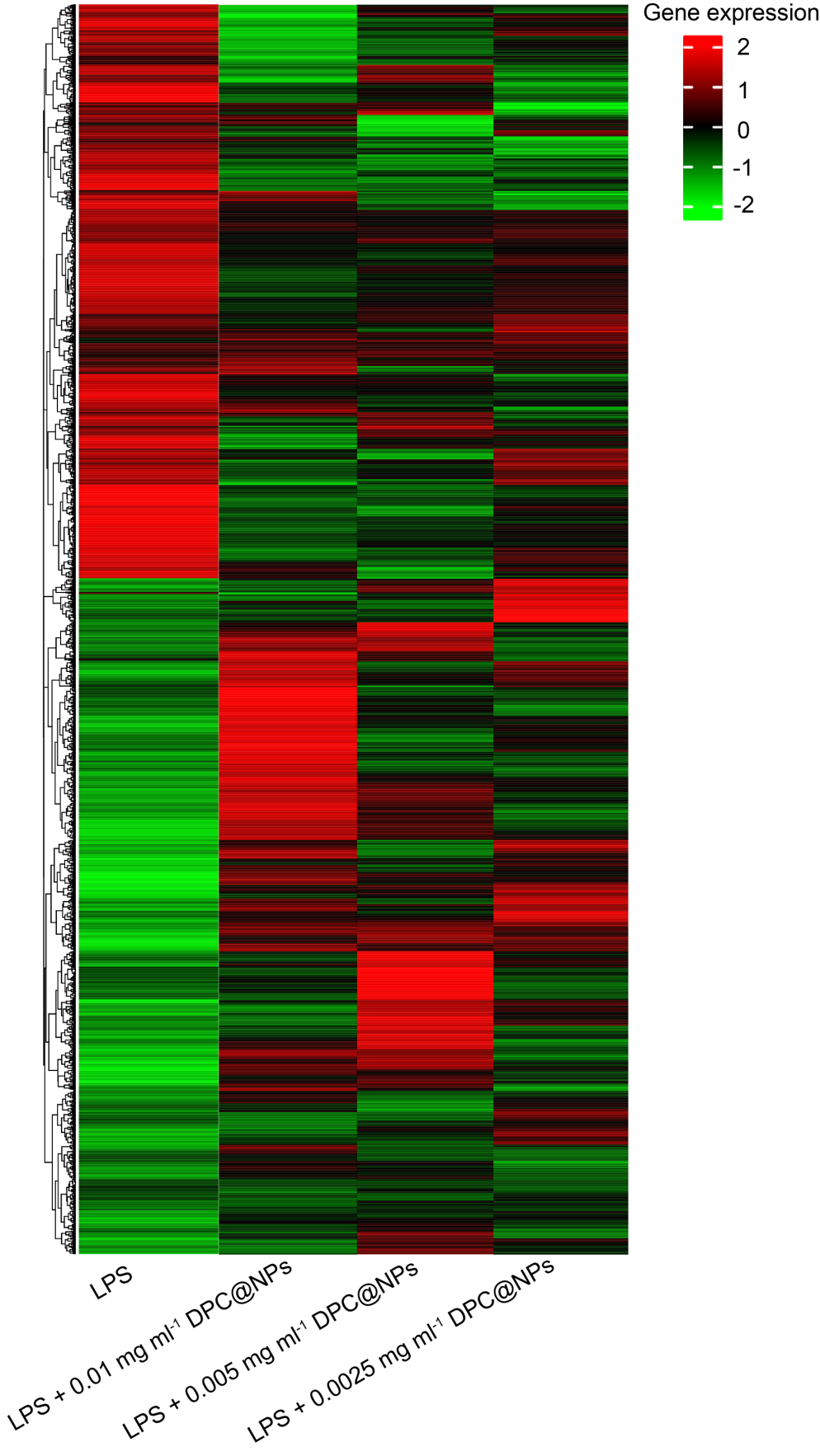


**Fig. S10** Heatmap of differentially expressed genes among LPS, LPS + 0.01 mg·mL^-1^ DPC@NPs, LPS + 0.005 mg·mL^-1^ DPC@NPs, and LPS + 0.002 5 mg·mL^-1^ DPC@NPs.


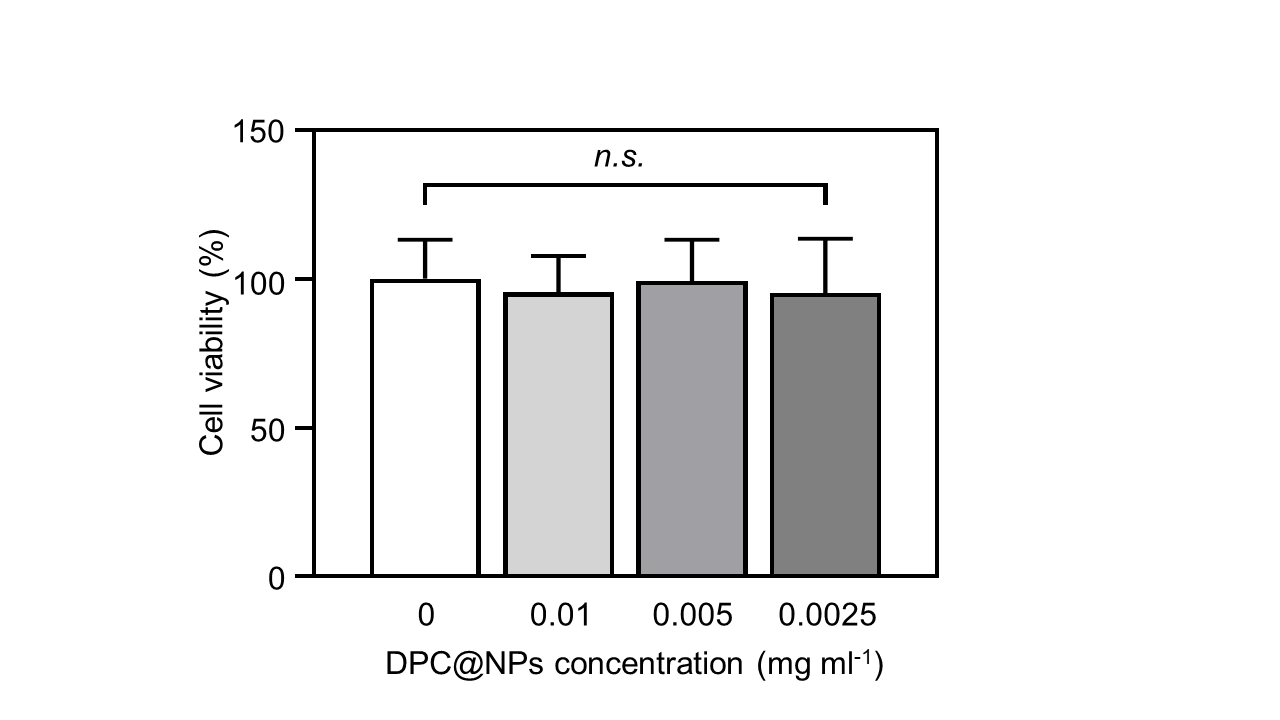


**Fig. S11** Cell viability of DPCs incubated with DPC@NPs at various concentrations (0.01, 0.005, and 0.002 5 mg·mL^-1^) for 24 h. *n.s.* represented no significance. Data presented as mean ± s.d. (n = 5).


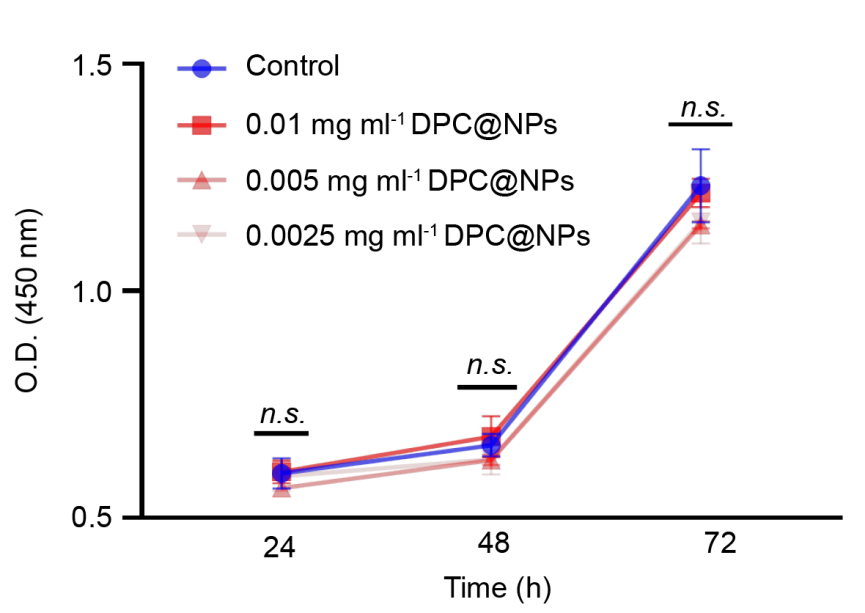


**Fig. S12** Proliferation of DPCs incubated with DPC@NPs at various concentrations (0.01, 0.005, and 0.002 5 mg·mL^-1^) for 24, 48, and 72 h, respectively. *n.s.* represented no significance. Data presented as mean ± s.d. (n = 5).


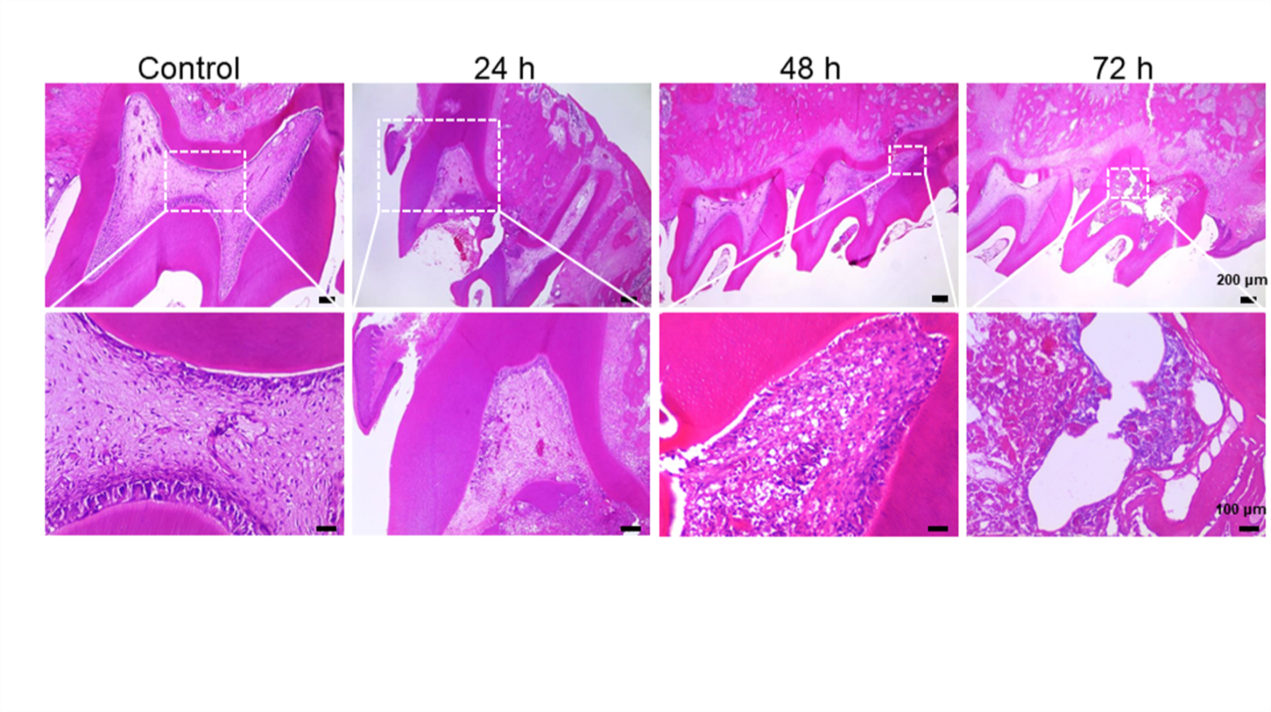


**Fig. S13** Images of slices stained with H&E. The dental pulp tissues were stimulated with 20 μL *E. coli* LPS (10 μg·mL^-1^) for 24, 48, and 72 h. Pulp tissues without the stimulation of *E. coli* LPS were used as a control.


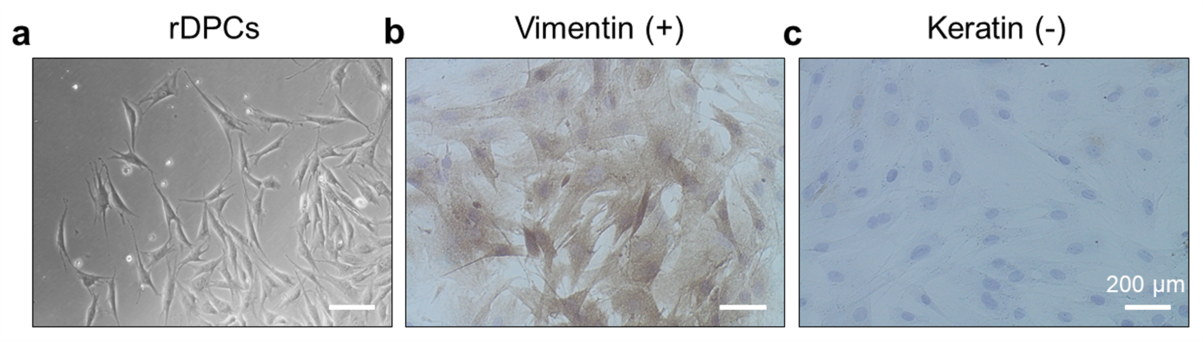


**Fig. S14** **a** Morphology of rDPCs. rDPCs were stained with **b** vimentin and **c** keratin by ICC. Scale bars = 200 μm.


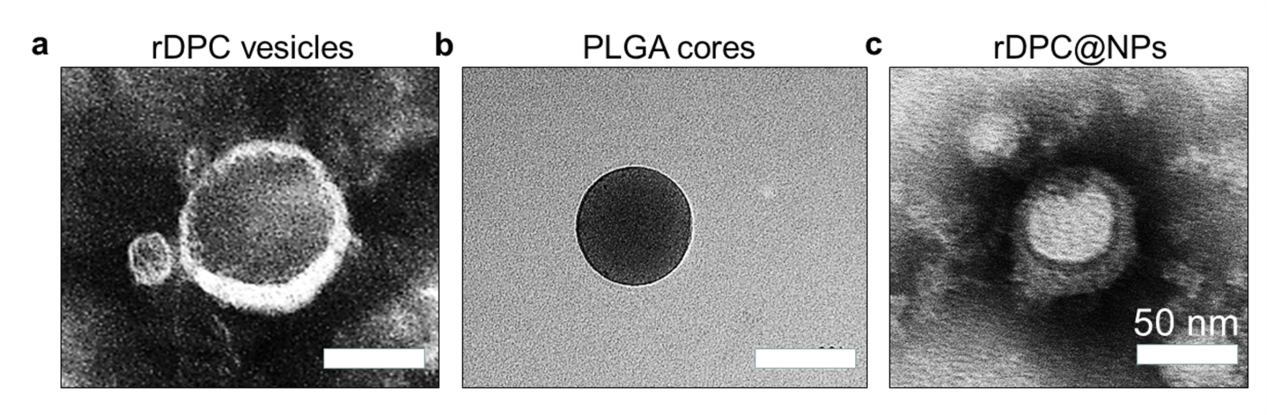


**Fig. S15** TEM images of **a** membrane-derived vesicles from rDPCs, **b** PLGA nanosphere, and **c** DPC@NPs.


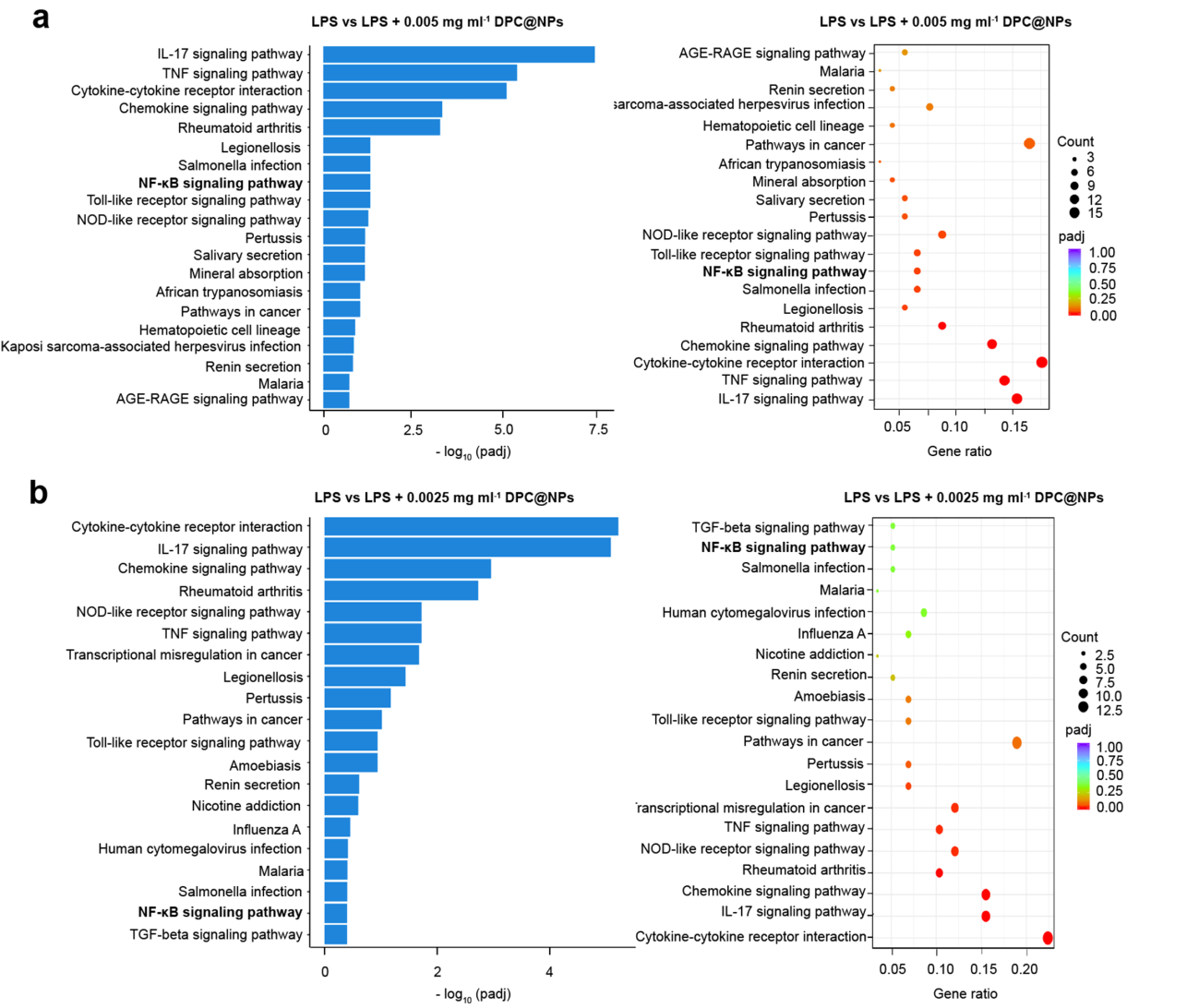


**Fig. S16** Changes of the top 20 enriched KEGG pathway terms between LPS and **a** LPS + 0.005 mg mL^-1^ DPC@NPs and **b** LPS + 0.002 5 mg·mL^-1^ DPC@NPs.


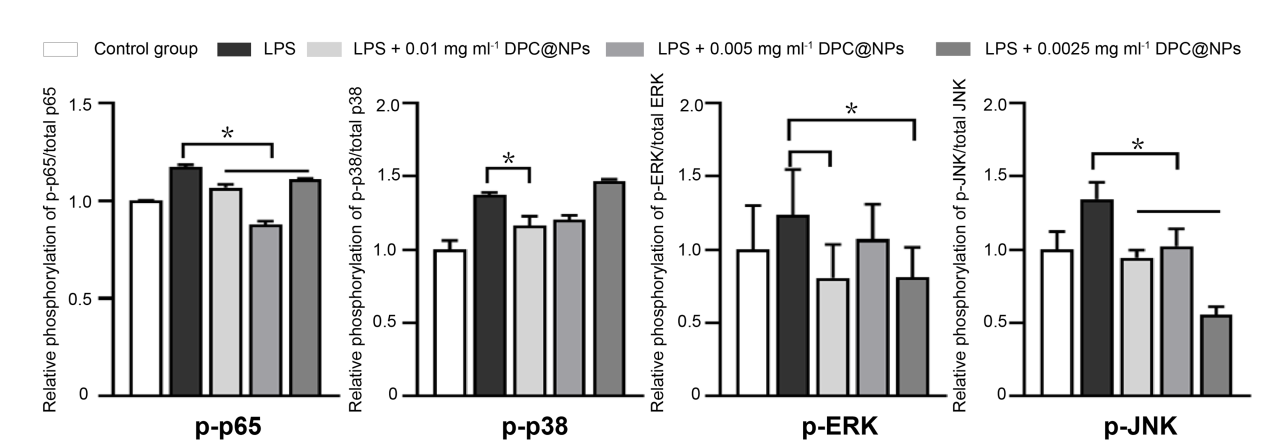


**Fig. S17** Quantification analysis of the relative phosphorylation of p-p65/total p65, p-p38/total p38, p-ERK/total ERK, and p-JNK/total JNK compared with the control group in WB.


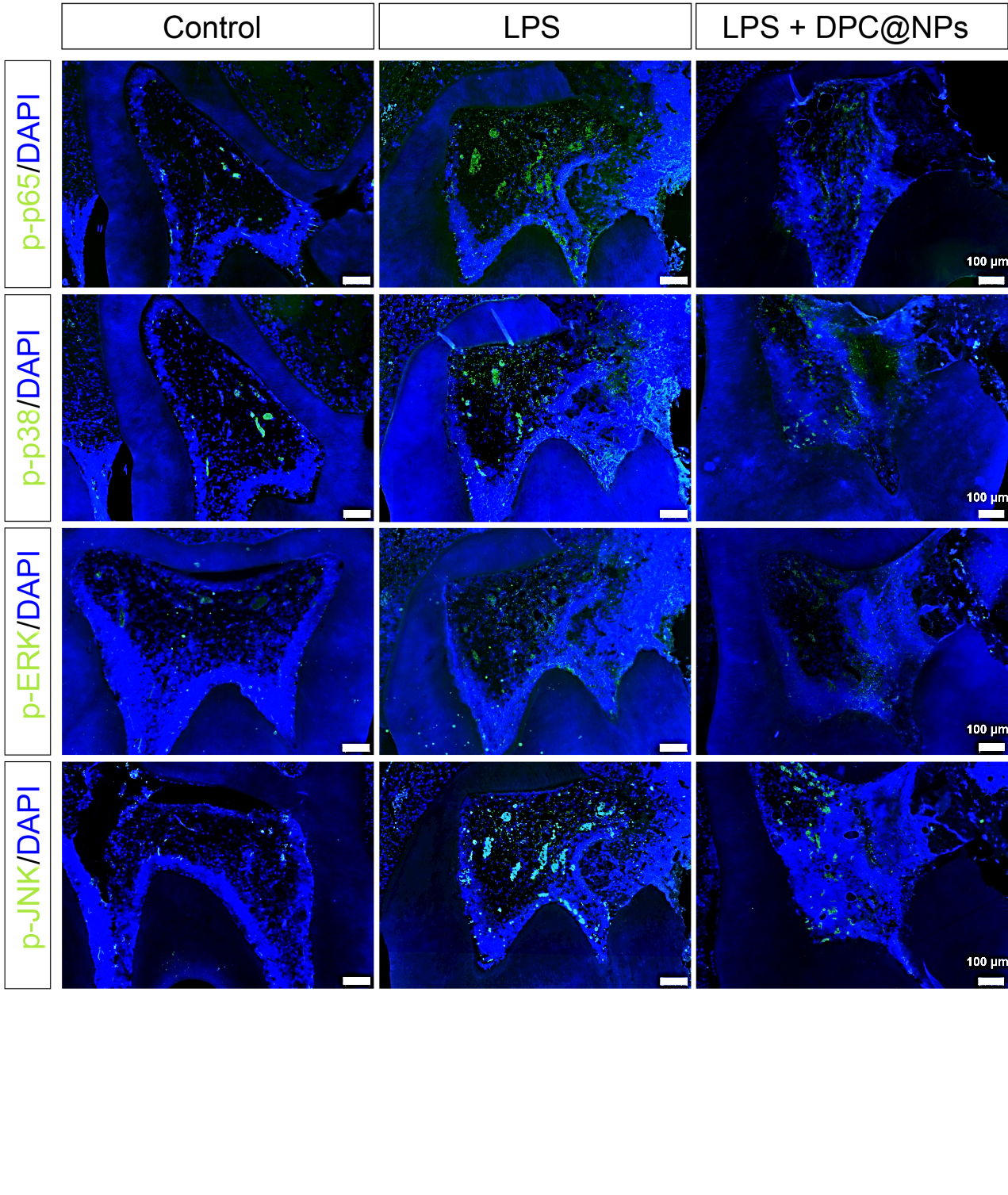


**Fig. S18** DPC@NPs suppressed the expression of p-p65, p-p38, p-ERK, and p-JNK *in vivo*. Each image was captured at magnifications of × 50*.*
